# Analysis of *bZIP* gene family in lotus (*Nelumbo*) and functional study of *NnbZIP36* in regulating anthocyanin synthesis

**DOI:** 10.1186/s12870-023-04425-2

**Published:** 2023-09-15

**Authors:** Ping Zhou, Jingwen Li, Huiyan Jiang, Qijiang Jin, Yanjie Wang, Yingchun Xu

**Affiliations:** 1 Key Laboratory of Landscaping, Ministry of Agriculture and Rural Affairs, Key Laboratory of Biology of Ornamental Plants in East China, College of Horticulture, Nanjing Agricultural University, Nanjing, 210095 Jiangsu China; 2 College of Horticulture, Nanjing Agricultural University, Nanjing, 210095 Jiangsu China

**Keywords:** Lotus, *bZIP* gene family, *NnbZIP36*, Anthocyanin, Gene cloning, Gene expression

## Abstract

**Background:**

The basic leucine zipper (bZIP) family is a predominant group of transcription factors in plants, involved in regulating plant growth, development, and response to stressors. Additionally, the *bZIP* gene family has a key role in anthocyanin production. Despite the significant role of *bZIP* genes in plants, their potential contribution in lotus remains understudied.

**Results:**

A total of 124 *bZIP* genes (59 *NnbZIPs* and 65 *NlbZIPs*) were identified from genomes of two lotus species. These genes were classified into 13 groups according to the grouping principle of the *Arabidopsis bZIP* gene family. Analysis of promoter *cis*-acting elements indicated that most *bZIP* gene family members in lotus are associated with response to abiotic stresses. The promoters of some *bZIP* genes contain MYB binding sites that regulate anthocyanin synthesis. We examined the anthocyanin content of the petals from three different colored lotus, combined with transcriptome data analysis and qRT-PCR results, showing that the expression trends of *NnbZIP36* and the homologous gene *NlbZIP38* were significantly correlated with the anthocyanin content in lotus petals. Furthermore, we found that overexpression of *NnbZIP36* in *Arabidopsis* promoted anthocyanin accumulation by upregulating the expression of genes (*4CL*, *CHI*, *CHS*, *F3H*, *F3'H*, *DFR*, *ANS* and *UF3GT*) related to anthocyanin synthesis.

**Conclusions:**

Our study enhances the understanding of the *bZIP* gene family in lotus and provides evidence for the role of *NnbZIP36* in regulating anthocyanin synthesis. This study also sets the stage for future investigations into the mechanism by which the *bZIP* gene family regulates anthocyanin biosynthesis in lotus.

**Supplementary Information:**

The online version contains supplementary material available at 10.1186/s12870-023-04425-2.

## Background

The basic region/leucine zipper motif (bZIP) transcription factor is one of the largest gene families of transcription factors, found in almost eukaryotes, and is highly conserved [1, 2]. The bZIP transcription factor consists of a basic region and a leucine zipper structure [3, 4], where the basic region is relatively conserved and consists of approximately 20 amino acid residues. The residues are able to bind to specific DNA sequences with an ACGT core such as a-box (TACGTA), C-box (GACGTC) and G-box (CACGTG) [3, 5]. The bZIP transcription factors play important roles in plant growth, development and the response to external environment, such as regulation of plant growth [6], flower development [7–9], seed maturation and dormancy [10], senescence [11], light signaling [12, 13], damage [14] and response to various environmental stresses [15–17].

With the development of high-throughput sequencing technology, a large amount of plant genomic data has been published and the *bZIP* gene family has been identified and studied in many plants, such as *Arabidopsis* [3, 18], rice (*Oryza sativa*) [19], soybean (*Glycine max*) [20], tomato (*Solanum lycopersicum*) [21], apple (*Malus domestica*) [22], etc. Involvement in response to various abiotic stresses/biotic stresses is a very typical and also the most reported function of bZIP transcription factors, such as salt stress, drought, high temperature, cold stress and pathogen infection [12, 19, 23]. For example, soybean contains 131 members of the *bZIP* gene family. More than 1/3 of them extensively involved in defense response including ABA, salt, drought, and cold stresses [24]. In *Arabidopsis*, AtbZIP17 and AtbZIP60 can enhance salt tolerance [25, 26]. AtbZIP1 can be promoted in *Arabidopsis* in response to abiotic stresses such as drought and high salt stresses [27]. Preeti et al. identified 191 bZIP transcription factors in wheat and found that TabZIPs were may play a role in various stress (high temperature, drought, salt stress) relief mechanisms. Overexpression of *TabZIPs* in *Arabidopsis* enhanced the ability of transgenic *Arabidopsis* to tolerate salinity, drought, high temperature, and oxidative stresses [28]. In recent years, with the in-depth study of bZIP transcription factors, it has been found that bZIPs are involved in regulating the synthesis of plant secondary metabolites in addition to responding to abiotic stresses and participating in the regulation of plant growth and development. It has been shown that some *bZIP* genes are involved in the regulation of anthocyanin synthesis, such as *HY5* (Elongated hypocotyl 5) and *HYH* (HY5 homolog) [29–31]. In *Arabidopsis*, *HY5* can promote anthocyanin accumulation by interacting with the promoters of some MYB transcription factors or directly with MYB transcription factors to promote the expression of genes related to anthocyanin synthesis [30]. Similarly, in tomato and apple, *HY5* has a similar function in the regulating anthocyanin biosynthesis [29, 31]. Tu et al. demonstrated that VvbZIP36 is a negative regulator of anthocyanin synthesis using CRISPR/Cas9 technology. VvbZIP36 plays a role in balancing the synthesis of stilbene (α-glucosin), lignans, flavonols and anthocyanins [32]. This evidence suggests that bZIP plays an important role in the regulation of the plant flavonoid synthesis pathway.

Lotus (*Nelumbo*) is one of the ten traditional Chinese flowers with high ornamental value [33]. Lotus are rich in flavonoid substances, which provide them with rich floral color and high medicinal value [34]. It is of great significance to study of flavonoids biosynthesis in lotus to improve the medicinal value and enrich the flower color of lotus. However, most of the reports related to anthocyanin biosynthesis of lotus have focused on MYB-bHlH-WD40 complex, while there is no report on the regulation of anthocyanins in lotus by bZIP. In this study, 59 *NnbZIP* genes and 65 *NlbZIP* genes were identified from the genomes of Asian lotus and American lotus, respectively. The chromosomal distribution, gene structure, conserved motifs and evolution of *bZIP* gene family were further investigated. The regulation of lotus *bZIP* genes on anthocyanin synthesis in lotus was also analyzed. The *NnbZIP36* gene was cloned as a representative *bZIP* gene, the regulation of anthocyanins by *NnbZIP36* was also studied by ectopic expression in *Arabidopsis*. These results provide a reference for studying the regulation of anthocyanin synthesis by the *bZIP* gene family in lotus, and provide a basis for further elucidating the mechanism of lotus flower color formation.

## Materials and methods

### Acquisition of sequencing data

Genomic data for the lotus of *Nelumbo nucifera* (*N. nucifera*) and *Nelumbo lutea* (*N. lutea*) used in this study are available in the databases (http://nelumbo.cngb.org/nelumbo/home) and NCBI (https://www.ncbi.nlm.nih.gov/) respectively under BioProject number PRJNA747731. The transcriptome data associated with this study were downloaded from the NCBI's SRA database (https://www.ncbi.nlm.nih.gov/sra/?term=) and the transcriptome data numbering information is shown in Table S1.

### Calculation of transcriptome gene expression

The transcriptome cleaned data were aligned to the lotus reference genome using hisat2 software [35–37]. Gene expression levels were calculated using the R package DESeq2 [38].

### Identification of *bZIP* genes in the genomes of *N. nucifera* and *N. lutea*

The Hidden Markov Models (HMM) files containing sequences of conserved domains of bZIP_1 (PF00170), bZIP_2 (PF07716), bZIP_C (PF12498), bZIP_Maf (PF03131), and HLH (PF00010) were downloaded from the Pfam database (http://pfam.xfam.org). Based on the Hidden Markov Model (HMM) files, the *bZIP* gene sequences of *N. nucifera* and *N. lutea* were queried using HMMER software. The *bZIP* genes in both genomes of lotus were further identified based on 79 AtbZIP protein sequences from *Arabidopsis* using BLASTp software. Conserved structural domain analysis of the bZIP protein sequences obtained was performed using the Simple Modular Architecture Research Tool (SMART: http://smart.embl-heidelberg.de) to ensure the validity of the identified *bZIP* genes.

### Characterization of the bZIP TFs in lotus

According to the gff file information of *N. nucifera* and *N. lutea* genomes, the length and position information of the lotus *bZIP* genes were extracted. Molecular weight (MW) and isoelectric point (pI) of the lotus *bIZP* genes were calculated using the ProtParam tool in ExPASy Server (https://web.expasy.org/protparam/). The information of the structure, location and conserved components of the *NnbZIPs* and *NlbZIPs* genes were examined using the MEME online tool, identifying conserved motifs shared by the bZIP proteins. The MAST xml files were downloaded and visualized using TBtools software. The chromosomal distribution and gene structures (including introns and utr) of NnbZIPs and NlbZIPs were analyzed using TBtools [39].

### Phylogenetic analysis of the NnbZIPs and NlbZIPs protein

The amino acid full-length sequences of bZIP proteins from seven species were aligned using muscle software with default parameters, and a neighbour-joining (NJ) phylogenetic tree was constructed using MEGA 7.0 software with a bootstrap replication number of 1000 [40]. The bZIP transcription factors were classified into different groups according to the topology of the phylogenetic tree.

In order to study the expression characteristics of *bZIP* genes in *N. nucifera* and *N. lutea*, the 2000 bp sequence upstream of the start codon (ATG) of *bZIP* genes were obtained as the promoter region, and the phylogenetic tree of the promoter sequence was constructed using MEGA 7.0 software with performed 1000 bootstrap replications.

### Analysis of the *cis*-acting element of the promoter of the *bZIP* genes in lotus

The obtained *bZIP* gene promoter sequences were uploaded to the PlantCARE database (http://bioinformatics.psb.ugent) for *cis*-acting regulatory element prediction. Visualized using TBtools based on the database analysis results.

### Identification of gene duplication patterns and covariance analysis

The MCScan (https://github.com/tanghaibao/jcvi/wiki/MCscan-Python) software was used to analyze the gene duplication events of NnbZIP and NlbZIP members [41], and the gene duplication relationship was visualized using TBtools software. The TBtools software was used to estimate the non-synonymous substitution rate (Ka), the synonymous substitution rate (Ks) and their ratio (Ka/Ks) [39].

The python version of MCscan (JCVI v1.1.7) [42] was used to perform a comparative analysis of the genomes of six plants. Co-linear modules with a co-linear relationship to the lotus *bZIP* genes were highlighted.

### Experiment material

The experimental materials used in this study were collected at Baima Base for Teaching and Scientific Research of Nanjing Agricultural University. The experimental material cultivars were: the ancient Chinese lotus and the American yellow lotus. The samples were frozen in liquid nitrogen and stored in a -80 °C refrigerator. Wild-type *Arabidopsis* plants were grown in incubators (*Arabidopsis* seeds were kept in our laboratory).

### RNA isolation, cDNA synthesis and quantitative real-time PCR analysis

The total RNA was extracted using the FastPure Universal Plant Total RNA Isolation Kit (Novazyme Biotechnology, China), and the experimental steps were referred to the kit instructions. The integrity of total RNA was detected by 1% agarose gel electrophoresis, and the concentration was determined using NanoDrop 1000 (Thermo, USA). The first-strand cDNA was synthesized using HiScript III RT SuperMix for qPCR (gDNA wiper) kit (Novazyme Biotechnology, China), and 1 μg of total RNA was used for each 20 μL reaction. The cDNA product was diluted tenfold with deionized water before use.

qRT-PCR experiments were performed using ChamQ SYBR qPCR Master Mix kit (Novozymes Biotechnology, China) on a CFX96 Touch™ Real Time PCR Detection System (BIORAD, USA). 2 μl of diluted cDNA was used for each reaction and other reaction components and conditions were carried out according to the manufacturer's instructions. Specific primers were designed for the qPCR (Table S2). The *Actin* gene of lotus [43] was used as an internal reference gene and was performed in three biological and three technical replicates of each treatment. Relative expression was calculated using the 2^−ΔΔCt^ method [44, 45].

### Gene cloning of *NnbZIP36* and its heterologous expression in *Arabidopsis*

The complete transcript sequence of the *NnbZIP36* gene was cloned using 2 × Hieff Canace® Plus PCR Master Mix (With Dye) (Yeasen Biotech Co.). PCR reactions were carried out in a volume of 50 μL under the following conditions: denaturation at 98 °C for 2 min, 40 thermal cycles (98 °C / 10 s; 60 °C / 20 s; 72 °C / 30 s) and a final extension at 72 °C for 5 min. The PCR products were separated by 1% agarose gel electrophoresis and the gel was cut to recover the target fragments. After recovery, ligation, transformation and sequencing, the final CDS sequence of the *NnbZIP36* gene was obtained.

The CDS sequence of the *NnbZIP36* gene was assembled in the overexpression vector pFAST-R05 using homologous recombination approach. The correct recombinant plasmid obtained by sequencing was transferred into *Agrobacterium tumefaciens GV3101* using freeze-thawing method. It was inoculated in 50 mL of LB liquid medium containing 100 mg/L kanamycine and 50 mg / L rifampicin, incubated at 200 r / min for 48 h at 28 °C, and then centrifuged at 4000 rpm for 10 min to collect the bacteria. Bacteria were resuspended with an osmotic agent (0.05% sliwet77 + 5% sucrose + 1/2 MS liquid medium). The *Arabidopsis* plants were subsequently transformed according to the flower dip method and incubated in the dark for 48 h. After three infestations, *Arabidopsis* seeds were collected and screened for *NnbZIP36* transgenic positive plants [46]. The positive plants were identified using PCR and qRT-PCR methods. Seeds of the identified positive plants were grown in 1/2 MS Petri dishes and positive seedlings with two true leaves were photographed after two weeks. The primers used for cloning and vector construction are listed in Table S2.

### Anthocyanin content determination

Total anthocyanin content (TAC) was determined by spectrophotometric method [47, 48]. The anthocyanins were extracted from 1 g sample with methanol (0.05% hydrochloric acid). The absorbance of the anthocyanins was measured at 510 nm and 700 nm using a spectrophotometer (UV -2550, Shimadzu, Japan). Absorbance (Abs) is calculated as: Abs = (A_510 nm_-A_700 nm_)pH_1.0_-(A_510 nm_-A_700 nm_)pH_4.5_ [47, 49]. Total anthocyanin content was calculated from the following formula: TAC ( %) = Abs /eL × MW × D × V/G × 100. ‘e’ represents the molar absorbance of anthocyanin 3-glucoside [26 900 ml (mmol cm)^−1^]. ‘L’ is the cell path length (1 cm). ‘MW’ is the molecular weight of anthocyanin (449.2 g mol^−1^). ‘D’ is the dilution factor. ‘V’ is the final volume (ml). ‘G’ is the mass of FW (g).

### Statistical analysis

Data in this study are shown as mean ± standard error (SE) of 3 or 6 independent biological replicates. Statistical differences between samples were analyzed by LSD and Duncan (D) (*p* < *0.05*). Data analysis and visualization were processed using SPSS 20.0 and GraphPad Prism 8.0.

## Results

### Identification of bZIP TFs in lotus

Fifty-nine *NnbZIP* genes and 65 *NlbZIP* genes were identified from *N. nucifera* and *N. lutea*, respectively. To facilitate subsequent analysis, the identified *bZIP* genes were named according to their chromosomal location (Table 1). The physicochemical properties of the lotus *bZIP* genes were analyzed using the ExPASy online database (Table 1). The length of the protein encoded by the *NnbZIP* genes ranges from 138 (NnbZIP34) to 885 aa (NnbZIP52), the molecular weight ranges from 15,808.92 (NnbZIP34) to 98,132.36 (NnbZIP52), and the theoretical isoelectric constant ranges from 5.02 (NnbZIP40) to 10.90 (NnbZIP43), the instability index ranges from 33.2 (NnbZIP54) to 85.97 (NnbZIP30), the aliphatic amino acid index ranges from 47.15 (NnbZIP32) to 89.15 (NnbZIP13), and the GRAVY ranged from − 1.235 (NnbZIP33) to − 0.08 (NnbZIP13). The length of the protein encoded by the *NlbZIP* genes ranges from 138 aa (NlbZIP35) to 884 aa (NlbZIP58), the molecular weight ranges from 15,794.89 (NlbZIP35) to 97,924.04 (NlbZIP58), and the theoretical isoelectric constant ranges from 4.63 (NlbZIP50) to 10.90 (NlbZIP47). The stability index ranges from 39.7 (NlbZIP52) to 75.54 (NlbZIP01), the aliphatic amino acid index ranges from 49.07 (NlbZIP33) to 88.92 (NlbZIP16), and the GRAVY ranges from − 1.213 (NlbZIP34) to − 0.089 (NlbZIP16). The *NnbZIP* and *NlbZIP* genes are distributed on all eight chromosomes of lotus, with relatively high numbers distributed on chr1, chr2, and chr5, and the least distributed on chr8 (Fig. S1).

**Table 1:** The detailed characteristics of *bZIP* genes identified in lotus

Species	Gene name	Gene ID	Chromosome location	Protein length (aa)	MW (Da)	pl	Instability Index	Aliphatic Index	Grand Average of Hydropathicity
Nelumbo nucifera	NnbZIP01	Nn1g01027.2	chr1:21,884,425–21,886,791(-)	428	48,400.16	8.49	65.87	66.12	-0.853
	NnbZIP02	Nn1g01783.5	chr1:38,024,045–38031157( +)	391	41,114.53	5.98	49.81	62.74	-0.671
	NnbZIP03	Nn1g01860.4	chr1:39,597,299–39,628,157( +)	413	43,573.84	5.79	64.83	52.78	-0.784
	NnbZIP04	Nn1g02540.2	chr1:53,032,933–53042459( +)	324	35,156.05	5.85	65.68	65.74	-0.712
	NnbZIP05	Nn1g04300.6	chr1:89,353,690–89355898(-)	213	23,624.4	5.57	66.66	68.69	-0.731
	NnbZIP06	Nn1g05081.2	chr1:104,743,042–104752148(-)	253	27,538.55	6.01	66.92	70.59	-0.725
	NnbZIP07	Nn1g05226.2	chr1:108,068,266–108097180(-)	404	45,226.15	5.65	53.32	82.87	-0.395
	NnbZIP08	Nn1g05228.1	chr1:108,130,910–108136774( +)	313	34,557.96	8.73	47.68	77	-0.579
	NnbZIP09	Nn1g05814.1	chr1:120,927,766–120928236(-)	156	17,701.74	6.42	70.73	75.77	-0.756
	NnbZIP10	Nn1g06071.8	chr1:126,758,084–126768794( +)	312	34,282.93	9.49	53.61	76.03	-0.594
	NnbZIP11	Nn1g06336.2	chr1:132,336,815–132,339,264(-)	236	26,429.46	5.87	65.4	66.1	-0.746
	NnbZIP12	Nn2g10316.2	chr2:1,843,266–1,848,587( +)	301	33,392.47	6.72	44.56	71.66	-0.579
	NnbZIP13	Nn2g10802.2	chr2:8,646,183–8,669,460( +)	843	92,717.88	5.97	50.13	89.15	-0.08
	NnbZIP14	Nn2g11976.3	chr2:26,204,638–26206497( +)	172	18,665.62	9.55	62.93	64.19	-1.072
	NnbZIP15	Nn2g14144.1	chr2:63,446,589–63,450,719( +)	478	52,539.2	8.06	49.17	60.27	-0.852
	NnbZIP16	Nn2g14147.1	chr2:63,582,105–63588973(-)	492	54,804.49	6.62	58.19	72.11	-0.558
	NnbZIP17	Nn2g14480.1	chr2:71,631,849–71,632,439( +)	196	21,702.15	7.94	52.46	63.21	-0.508
	NnbZIP18	Nn2g14613.1	chr2:76,716,889–76,741,219(-)	342	37,714.44	7.79	59.59	67.05	-0.831
	NnbZIP19	Nn2g15149.1	chr2:97,251,867–97,252,724(-)	285	31,338.08	6.41	37.18	77.26	-0.651
	NnbZIP20	Nn2g15572.1	chr2:105,269,822–105275282(-)	450	50,320.06	8.47	61.77	75.93	-0.515
	NnbZIP21	Nn3g17590.1	chr3:20,626,366–20626845( +)	159	18,627.98	7.89	69.43	66.29	-0.951
	NnbZIP22	Nn3g17787.1	chr3:23,237,163–23,246,563( +)	454	50,236.19	5.87	39.63	80.37	-0.402
	NnbZIP23	Nn3g18441.3	chr3:32,780,275–32818901( +)	473	51,687.06	7.62	58.66	62.75	-0.504
	NnbZIP24	Nn3g18937.2	chr3:41,293,105–41310530(-)	373	42,354.03	8.47	77.79	67	-0.971
	NnbZIP25	Nn3g19377.1	chr3:48,821,070–48836462( +)	419	44,732.38	6.03	59.86	57.3	-0.707
	NnbZIP26	Nn3g19494.1	chr3:50,973,969–50994667(-)	455	48,727.79	5.21	69.55	60.53	-0.708
	NnbZIP27	Nn3g20228.2	chr3:74,026,818–74036432(-)	846	92,666.78	6.07	47.53	86.37	-0.145
	NnbZIP28	Nn3g21690.1	chr3:107,140,650–107141087( +)	145	16,776.04	6.59	61.31	81.31	-0.741
	NnbZIP29	Nn4g23591.1	chr4:23,522,215–23,522,811( +)	198	22,885.61	6.07	78.99	79.34	-0.855
	NnbZIP30	Nn4g23593.1	chr4:23,549,051–23549524( +)	157	18,224.51	9.68	85.97	70.25	-0.941
	NnbZIP31	Nn4g23777.4	chr4:26,449,238–26,457,366( +)	453	50,049.82	6.07	47.03	75.81	-0.489
	NnbZIP32	Nn4g24488.4	chr4:38,930,123–38947958( +)	376	39,607.22	5.96	58.47	47.15	-0.902
	NnbZIP33	Nn4g25307.3	chr4:63,610,135–63614948( +)	155	17,819.76	9.69	70.22	64.26	-1.235
	NnbZIP34	Nn4g25598.1	chr4:72,295,289–72,295,705(-)	138	15,808.92	9.26	68.69	82.03	-0.724
	NnbZIP35	Nn5g26856.1	chr5:3,237,159–3,241,883( +)	378	41,186.76	5.81	61.72	66.43	-0.68
	NnbZIP36	Nn5g28704.4	chr5:49,839,872–49,846,207(-)	289	31,996.91	6.62	48.03	61.49	-0.764
	NnbZIP37	Nn5g28705.2	chr5:49,891,020–49893229( +)	237	26,029.99	6.01	49.31	56.41	-0.741
	NnbZIP38	Nn5g28759.1	chr5:50,647,221–50656919(-)	290	31,529.26	6.1	53.19	74.72	-0.506
	NnbZIP39	Nn5g28963.1	chr5:53,834,875–53,838,494( +)	318	35,069.53	8.73	46.57	79.37	-0.554
	NnbZIP40	Nn5g29527.3	chr5:62,547,999–62,565,826( +)	392	41,840.68	5.02	59.05	54.08	-0.835
	NnbZIP41	Nn5g29701.1	chr5:65,155,446–65,155,907( +)	153	17,474.47	5.79	71.02	77.84	-0.729
	NnbZIP42	Nn5g30287.4	chr5:73,595,442–73,603,126(-)	446	47,749.51	9.45	47.11	66.05	-0.607
	NnbZIP43	Nn5g30657.1	chr5:78,742,005–78742478( +)	157	18,615.94	10.9	55.21	69.49	-1.081
	NnbZIP44	Nn5g30783.3	chr5:80,909,384–80913748( +)	275	29,809.56	6.21	36.97	69.2	-0.591
	NnbZIP45	Nn6g31596.4	chr6:2,044,086–2048288( +)	303	34,451	5.05	41.67	80.43	-0.552
	NnbZIP46	Nn6g31991.1	chr6:9,133,148–9,136,487(-)	273	29,598.13	6.07	39.81	70.81	-0.636
	NnbZIP47	Nn6g32268.8	chr6:15,336,976–15,341,318(-)	526	57,136.81	6.52	67.18	54.62	-0.855
	NnbZIP48	Nn6g33807.1	chr6:37,958,370–37959471(-)	246	28,104.05	9.96	55.95	78.86	-0.664
	NnbZIP49	Nn6g34519.1	chr6:50,516,281–50516793( +)	170	19,856.45	9.91	64.53	79.71	-0.888
	NnbZIP50	Nn6g34689.1	chr6:53,468,972–53,477,830( +)	270	29,714.7	8.7	47.2	86.78	-0.494
	NnbZIP51	Nn6g34921.2	chr6:56,914,796–56,918,573(-)	400	43,529.52	8.42	49.27	65.62	-0.699
	NnbZIP52	Nn6g34983.2	chr6:57,690,499–57711800(-)	885	98,132.36	9.45	64.51	70.05	-0.735
	NnbZIP53	Nn7g36064.4	chr7:3,987,262–3,994,532( +)	428	47,877.51	7.68	58.19	81.24	-0.487
	NnbZIP54	Nn7g36371.1	chr7:8,858,533–8,859,357( +)	274	30,131.81	5.99	33.2	72.96	-0.577
	NnbZIP55	Nn7g36753.6	chr7:14,879,563–14,903,827( +)	419	46,206.81	7.14	53.58	74.06	-0.588
	NnbZIP56	Nn7g37256.1	chr7:24,051,516–24075199(-)	382	42,605.12	8.38	51.14	72.25	-0.728
	NnbZIP57	Nn7g37373.4	chr7:26,287,376–26,293,840( +)	358	38,229.2	5.83	59.47	57.32	-0.822
	NnbZIP58	Nn7g37592.1	chr7:31,749,123–31,766,461(-)	368	40,595.04	6.23	57.84	56.85	-0.882
	NnbZIP59	Nn8g39399.1	chr8:12,890,656–12893557( +)	185	20,290.52	9.09	55.98	76.54	-0.876
Nelumbo lutea	NlbZIP01	Al25565	chr1:26,597,497–26,599,746(-)	385	43,792.96	8.81	75.54	64.13	-0.917
	NlbZIP02	Al20307	chr1:52,972,663–52,983,305( +)	462	49,784.05	5.83	64.52	62.34	-0.74
	NlbZIP03	Al16253	chr1:83,238,172–83,249,097( +)	240	26,613.62	6.6	54.8	70.71	-0.717
	NlbZIP04	Al00810	chr1:90,594,032–90596023( +)	291	32,637.58	6.72	59.63	70.76	-0.82
	NlbZIP05	Al11844	chr1:90,831,120–90832781( +)	266	29,798.33	6.4	63.29	72.97	-0.796
	NlbZIP06	Al19863	chr1:102,491,551–102497845(-)	464	50,155.2	9.78	46.35	59.96	-0.759
	NlbZIP07	Al00283	chr1:107,462,717–107478100(-)	409	43,206.09	6.04	62.8	52.84	-0.86
	NlbZIP08	Al32391	chr1:109,127,425–109134250(-)	353	39,237.63	9.14	46.57	80.14	-0.478
	NlbZIP09	Al32390	chr1:109,167,968–109192360( +)	438	49,256.98	5.66	51.09	88.68	-0.281
	NlbZIP10	Al27610	chr1:122,443,033–122444822( +)	156	17,733.8	6.42	70.73	73.91	-0.771
	NlbZIP11	Al27102	chr1:128,949,323–128,959,405( +)	312	34,355.19	9.71	49.38	76.63	-0.567
	NlbZIP12	Al01268	chr1:135,319,042–135321132( +)	297	33,107.05	6.67	63.95	67.31	-0.866
	NlbZIP13	Al30011	chr1:165,275,919–165,280,725(-)	202	22,393.36	10.09	53.57	67.67	-0.726
	NlbZIP14	Al35379	chr1:214,448,215–214,458,724( +)	427	46,707.61	6.17	66.1	60.44	-0.827
	NlbZIP15	Al22101	chr2:2,665,621–2,678,243( +)	392	42,809.77	5.84	58.04	72.45	-0.603
	NlbZIP16	Al32080	chr2:11,446,406–11470846( +)	844	92,872.11	6.03	50.47	88.92	-0.089
	NlbZIP17	Al05492	chr2:27,215,741–27,217,978( +)	168	18,177.02	9.43	62.7	65.71	-1.061
	NlbZIP18	Al31819	chr2:43,295,533–43,302,155(-)	406	43,961.44	5.47	60.95	69.98	-0.655
	NlbZIP19	Al15367	chr2:50,173,470–50178107( +)	310	35,440.97	9.37	74.38	74.87	-0.879
	NlbZIP20	Al04258	chr2:63,133,285–63,139,305( +)	537	59,508.97	8.46	51.6	67.84	-0.734
	NlbZIP21	Al04195	chr2:64,471,837–64,489,063( +)	524	58,069.75	6.71	45.19	66.34	-0.735
	NlbZIP22	Al04190	chr2:64,602,750–64610458(-)	491	54,610.26	6.62	58.58	72.85	-0.556
	NlbZIP23	Al19970	chr2:86,236,594–86,239,829(-)	331	36,569.35	9.04	54.6	71	-0.772
	NlbZIP24	Al02500	chr2:87,015,216–87094362( +)	851	92,539.81	8.62	44.66	84.84	-0.228
	NlbZIP25	Al09426	chr2:106,513,142–106518447(-)	450	50,359.18	8.52	62.21	76.36	-0.496
	NlbZIP26	Al22667	chr3:23,423,756–23,432,397( +)	454	50,101.99	5.97	42.03	79.1	-0.416
	NlbZIP27	Al36235	chr3:31,959,861–31,998,921( +)	378	40,169.6	8.17	60.2	55.58	-0.717
	NlbZIP28	Al17761	chr3:47,909,057–47926950( +)	410	43,515.06	6.33	60.33	58.1	-0.691
	NlbZIP29	Al17878	chr3:50,057,420–50072109(-)	451	48,363.34	5.15	66.74	60.62	-0.713
	NlbZIP30	Al22201	chr3:76,110,320–76120041(-)	846	92,723.89	6.07	48.19	86.13	-0.148
	NlbZIP31	Al25392	chr3:109,744,333–109745928( +)	145	16,836.14	6.59	57.76	81.31	-0.716
	NlbZIP32	Al24783	chr4:25,862,750–25870163( +)	453	50,059.85	6.19	46.6	76.47	-0.491
	NlbZIP33	Al24184	chr4:37,861,464–37,887,762( +)	407	42,930.09	6.17	58.3	49.07	-0.842
	NlbZIP34	Al05079	chr4:62,653,456–62,658,128(-)	156	17,905.89	9.77	72.31	65.06	-1.213
	NlbZIP35	Al26010	chr4:73,399,965–73,401,280(-)	138	15,794.89	9.26	68.69	80.65	-0.741
	NlbZIP36	Al34789	chr5:3,422,706–3428050( +)	378	41,215.76	5.87	60.96	65.13	-0.683
	NlbZIP37	Al26493	chr5:28,358,109–28394521( +)	458	52,304.44	9.43	72.26	63.93	-1.013
	NlbZIP38	Al31480	chr5:54,588,906–54594819(-)	364	40,238.65	7.24	54.26	57.64	-0.895
	NlbZIP39	Al31481	chr5:54,624,573–54,628,920( +)	359	39,686.12	6.28	53.05	59.81	-0.823
	NlbZIP40	Al31526	chr5:55,411,225–55,420,912(-)	290	31,864.81	8.88	56.43	74.69	-0.606
	NlbZIP41	Al31705	chr5:58,428,520–58445151(-)	361	40,646.08	7.03	55.94	81.11	-0.434
	NlbZIP42	Al31709	chr5:58,504,504–58508151( +)	318	35,035.51	8.73	47.04	80.6	-0.551
	NlbZIP43	Al03730	chr5:61,133,814–61,138,602( +)	600	66,100.97	6.42	66.43	54.7	-0.95
	NlbZIP44	Al02680	chr5:65,718,591–65,738,021( +)	400	42,350.08	5.2	58.97	50.83	-0.878
	NlbZIP45	Al14243	chr5:68,239,523–68,241,045( +)	153	17,447.45	5.79	73.34	77.84	-0.712
	NlbZIP46	Al17524	chr5:76,144,065–76151258(-)	424	45,461.86	9.3	47.62	63.07	-0.684
	NlbZIP47	Al25335	chr5:81,485,652–81,486,125( +)	157	18,601.92	10.9	60.99	69.49	-1.082
	NlbZIP48	Al10012	chr5:83,695,244–83,698,802( +)	279	30,450.22	5.41	40.05	70.61	-0.587
	NlbZIP49	Al09994	chr5:83,937,019–83938120(-)	277	30,353.53	7.02	63.26	59.24	-0.681
	NlbZIP50	Al16974	chr6:1,185,565–1,191,068( +)	300	33,997.12	4.63	52.29	87.43	-0.616
	NlbZIP51	Al07183	chr6:7,211,711–7,212,901( +)	283	30,923.77	9.04	59.18	71.02	-0.351
	NlbZIP52	Al07162	chr6:7,681,689–7,684,913(-)	273	29,575.09	6.01	39.7	70.81	-0.637
	NlbZIP53	Al10496	chr6:16,153,870–16158692(-)	598	65,314.73	6.16	69.85	54.57	-0.964
	NlbZIP54	Al12576	chr6:51,146,713–51,147,524( +)	165	19,368.98	9.91	67.69	80.91	-0.871
	NlbZIP55	Al30745	chr6:54,678,437–54,687,275( +)	308	34,037.44	8.53	46.06	83.34	-0.531
	NlbZIP56	Al01743	chr6:57,501,613–57508667( +)	488	54,307.05	8.49	54.64	77.46	-0.547
	NlbZIP57	Al01748	chr6:57,645,562–57,650,759(-)	432	46,995.65	9.05	49.6	63.94	-0.723
	NlbZIP58	Al32784	chr6:58,547,140–58565087(-)	884	97,924.04	9.34	63.88	68.93	-0.733
	NlbZIP59	Al17163	chr7:7,849,991–7,855,640( +)	428	47,751.44	8.44	57.75	81.47	-0.471
	NlbZIP60	Al27256	chr7:22,158,671–22,163,517( +)	782	83,875.72	5.92	55.16	72.31	-0.506
	NlbZIP61	Al01891	chr7:29,415,590–29438800(-)	360	39,655.84	8.94	59.59	69.17	-0.754
	NlbZIP62	Al20570	chr7:31,529,838–31,538,198( +)	408	43,218.07	6.74	54.82	61.76	-0.664
	NlbZIP63	Al26110	chr7:36,478,932–36,505,235(-)	414	45,497.37	6.31	55.75	59.25	-0.845
	NlbZIP64	Al13878	chr8:14,395,166–14,398,040( +)	170	18,586.46	8.96	57.01	71.24	-1.056
	NlbZIP65	Al08728	chr8:49,293,649–49,298,708(-)	386	42,216.28	4.99	48.13	67.56	-0.707

### Phylogenetic classification and analysis of bZIP TFs in lotus

**Fig. 1:**
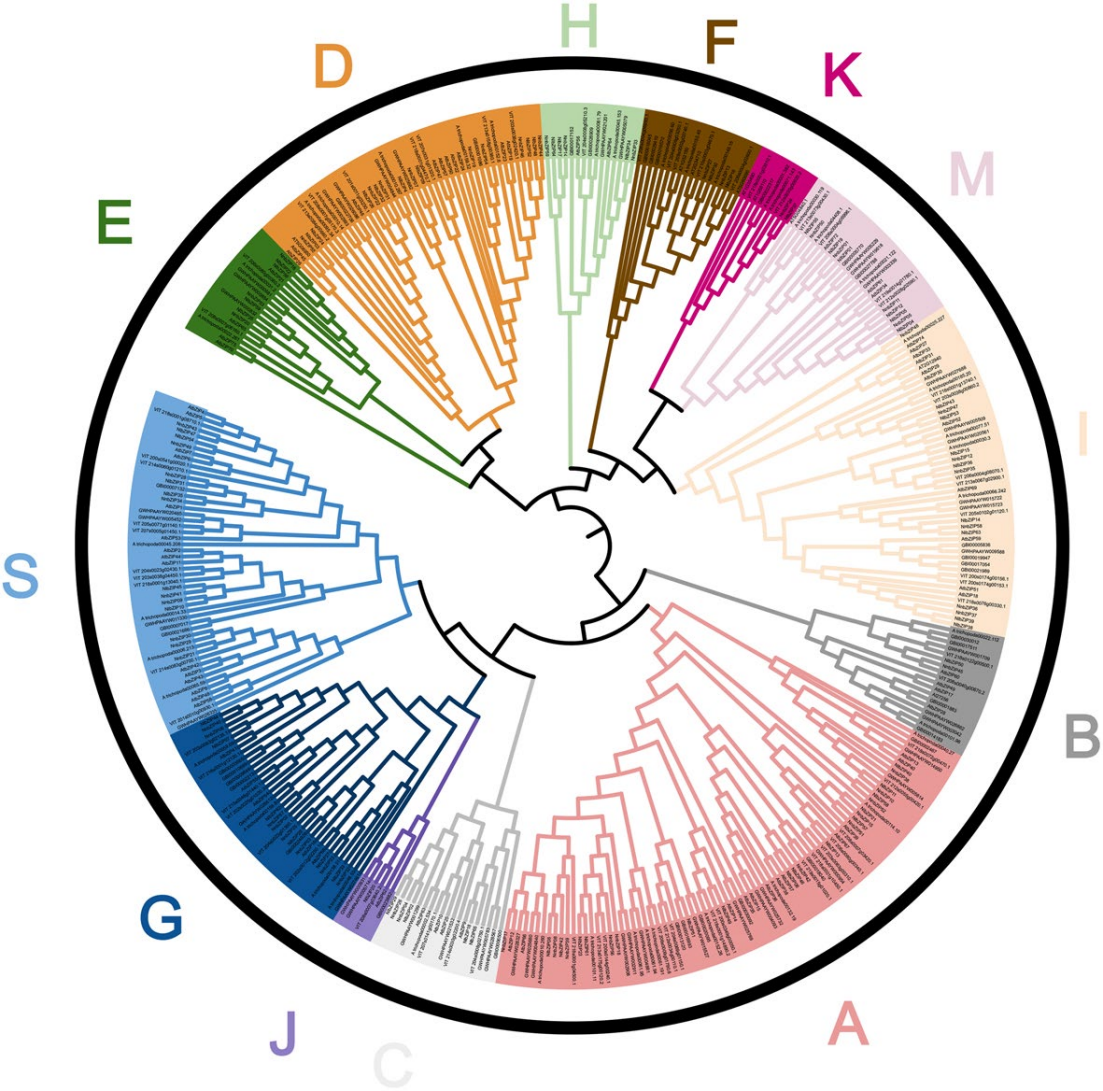
Phylogenetic tree of *bZIP* genes in seven species. The protein sequences of the bZIP TFs were compared using the maximum likelihood (ML) method. These proteins were divided into 12 groups and each group was assigned a different color

The bZIP protein sequences of two species of lotus, *Ginkgo*, *Amborella*, *Nymphaea colorata*, *Vitis vinifera* and *Arabidopsis* were aligned and a phylogenetic tree was constructed using MEGA 7.0 software. The bZIP proteins were classified into 13 groups (A, B, C, D, E, F, G, H, I, J, K, M and S) according to the grouping rules of *Arabidopsis bZIP* gene family (Fig. 1). The number of *bZIP* genes distributed among the 13 groups are differed considerably, with groups A, D, I and S possessing a higher number of *bZIP* genes. Group J had the fewest members with only six members, and the *bZIP* members of *N. nucifera* and *Amborella* were present. In addition, the *bZIP* gene families of these seven species were distributed in the remaining 12 groups. The results showed that most of the *NnbZIP* genes and *NlbZIP* genes have a high degree of similarity. However, there are still *NnbZIP* genes and *NlbZIP* genes that specific to each other. It indicated that the *N. nucifera* and *N. lutea* have evolved new *bZIP* genes after a long period of isolation in order to adapt to the local natural environment.

### Structural analysis of the *bZIP* gene family in lotus

**Fig. 2:**
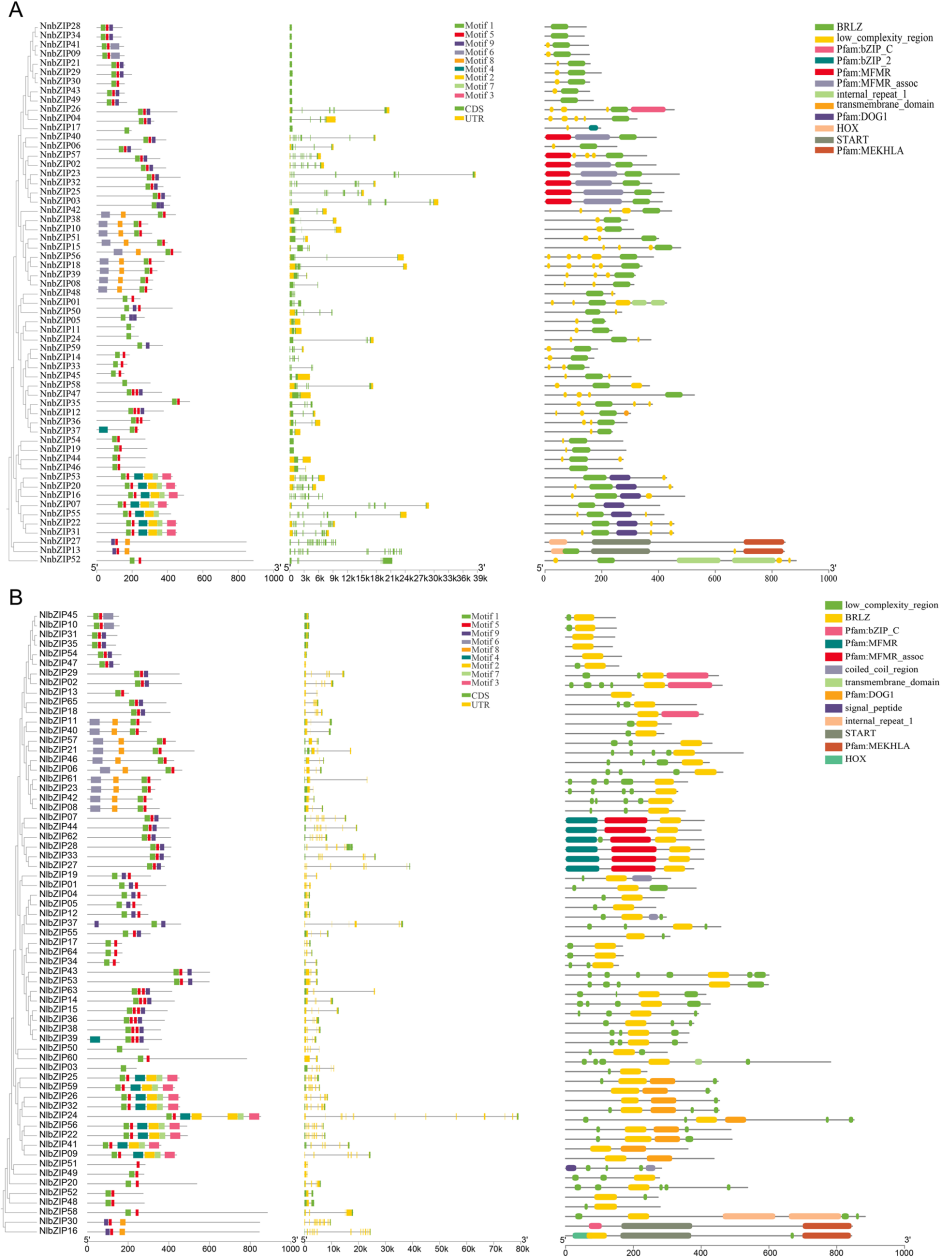
Gene structure analysis of the *bZIP* gene family in the lotus. **A**: Structure of the bZIP gene family in *N. nucifera*. **B**: Structure of the bZIP gene family in *N. lutea*. Exons and introns are shown using green bars and grey lines. bZIP members in protein motifs. The colored boxes depict the different patterns. Clustering is based on the results of phylogenetic analysis

Gene structure is an important reference information for measuring the evolution of gene families which can further support the results of phylogenetic trees [50]. Genetic structure analysis showed that most of the genes of *NnbZIP* and *NlbZIP* were structurally similar in the phylogenetic tree. Only a small number of *NnbZIP* and *NlbZIP* genes had large differences between them, and these genes are specific to the presence of *N. nucifera* and *N. lutea*, respectively (Fig. 2). In addition, bZIPs can be divided into two types according to the presence or absence of introns. Here, a total of 18 *bZIP* genes have no introns. 15 of the 18 *bZIP* genes are classified within group S, while *NnbZIP17*, *NnbZIP19* and *NnbZIP54* are classified in group E.

The protein conserved motif analysis showed that there were 9 conserved motifs in the *bZIP* gene family in lotus. Motif5 is the most conserved motif and is present in all *bZIP* genes. Furthermore, Motif1 was present in all but NnbZIP13, NnbZIP17, NnbZIP27, NlbZIP16 and NlbZIP30. It is speculated that Motif1 and Motif5 may be associated with conserved structural domains of the *bZIP* gene family. The conserved structural domains of the lotus *bZIP* gene family were identified using the SMART database. Model analysis showed that the *bZIP* gene family has three types of conserved structural domains, with the largest number of *bZIP* gene members possessing the BRLZ structural domain. The bZIP_C structural domain contains 4 members (NnbZIP13, NnbZIP27, NlbZIP16 and NlbZIP30). The bZIP_2 structural domain only has one member, NnbZIP17 (Fig. 2).

### The *cis*-acting elements of the *bZIP* gene promoter in lotus

**Fig. 3:**
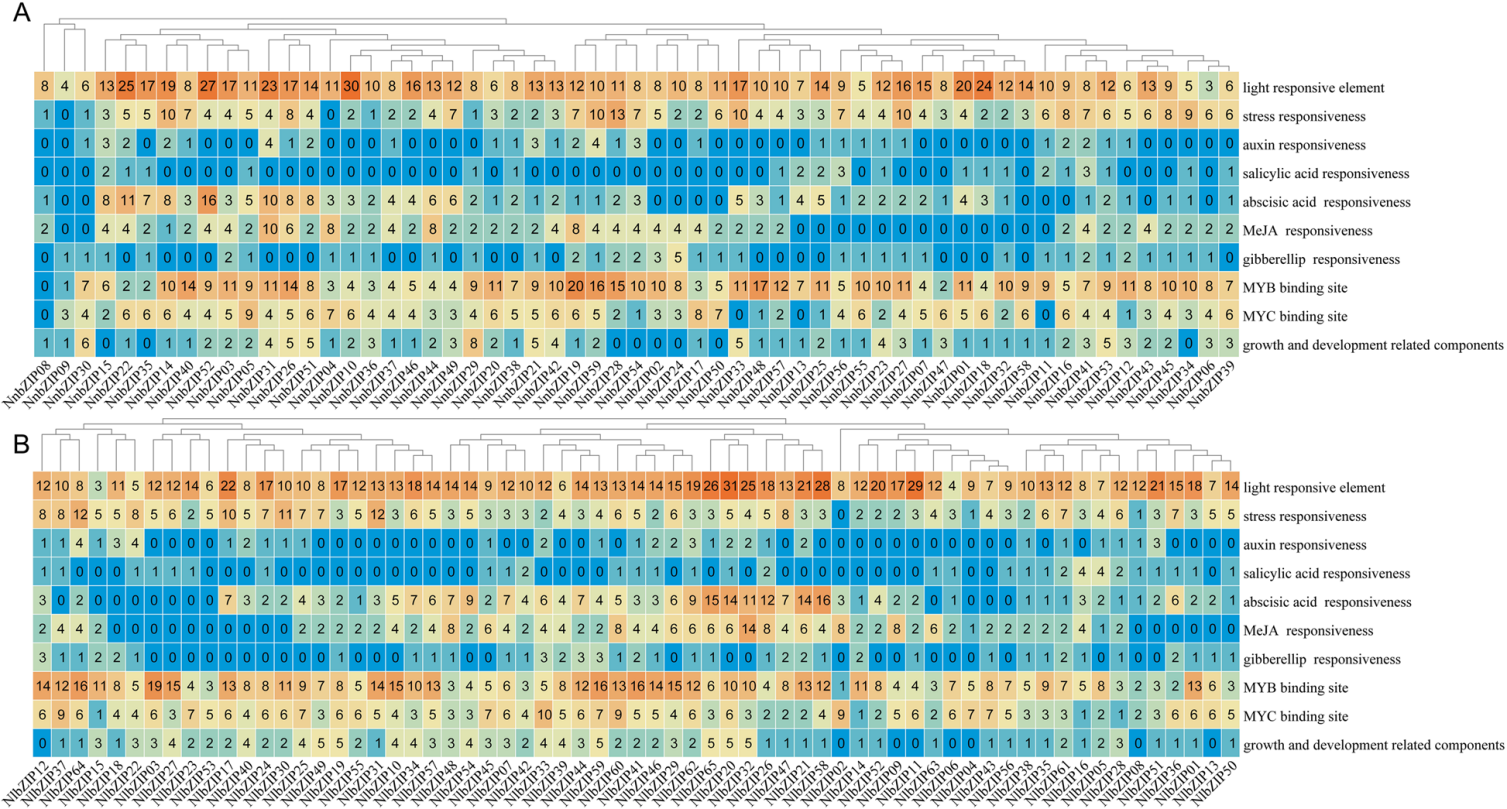
*Cis*-acting elements of the promoter region of *bZIP* genes in lotus. **A**: Number of different classes of cis-acting elements in the promoter region of the *N. lutea NlbZIP* genes. **B**: Number of different classes of cis-acting elements in the promoter of the *N. nucifera NnbZIP* genes

Prediction and analysis of *cis*-acting elements in gene promoters can help predict their function [51]. To understand the function of the *bZIP* gene family of lotus, the 2000 bp sequences upstream of the ATG of 124 *bZIP* genes (59 *NnbZIPs* and 65 *NlbZIPs*) were analyzed and a large number of *cis*-acting elements were identified (Fig. S2). In order to facilitate further analysis, these *cis*-acting elements were divided into four categories: light response, stress response, hormone response, growth and development-related elements (Fig. 3). Among them, light response elements were widely distributed in the promoters of all *bZIP* genes. With the exception of *NlbZIP02* and *NnbZIP09*, stress response-related elements are present in the promoters of all remaining *bZIP* genes. And they are generally more numerous compared to growth and development-related elements. This indicated that the *bZIP* gene family of lotus may play an important role in stress resistance of lotus. In addition, hormone response-related elements are also widely present in the promoter of the lotus *bZIP* genes. Interestingly, 9 *NlbZIP* genes and 8 *NnbZIP* genes have a large number of ABA-responsive elements in their promoters, so we speculate that these genes have similar functions to ABA-responsive factors (e.g. ABI3, ABI5, etc.). The large distribution of MYB-binding sites and MYC-binding sites also enhances the role of the *bZIP* genes in regulating the growth and development of the lotus and coping with the external natural environment. In conclusion, our results suggested that the lotus *bZIP* genes may respond to different signaling pathways through different types of *cis*-acting elements within their promoter regions.

### Collinearity analysis of *bZIP* genes in lotus

**Fig. 4:**
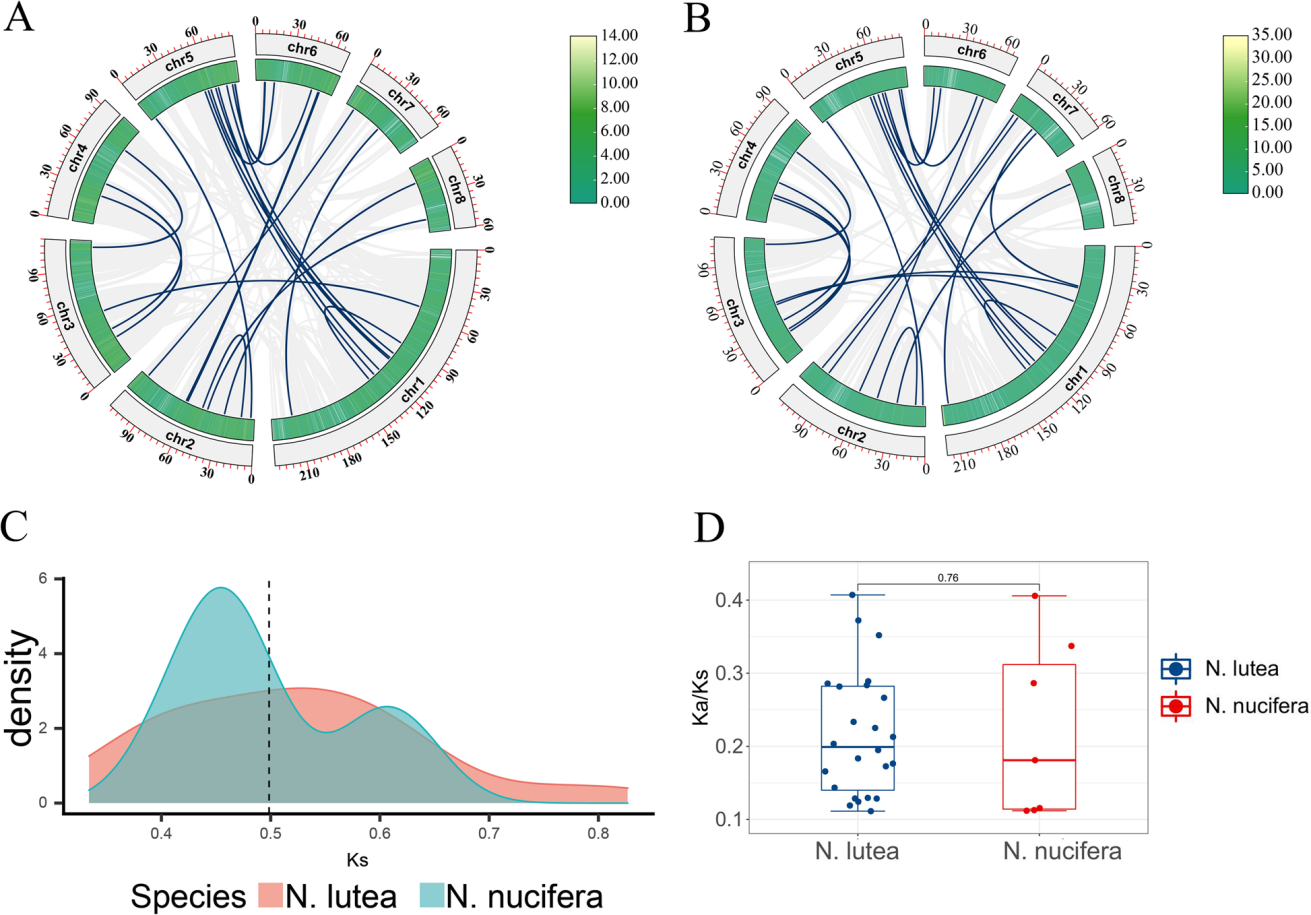
Covariance analysis and Ks distribution of *bZIP* replication genes in lotus. **A**: Covariance analysis of *bZIP* replication genes in *N. lutea.* **B**: Covariance analysis of *bZIP* replication genes in *N. nucifera*. **C**: Ks distribution of *bZIP* replication genes in two lotus species*.* **D**: Ka/Ks ratios of the two lotus species

MCScanX software was used to analyze the gene duplication events of *NlbZIP* genes and *NnbZIP* genes. A total of 24 gene duplication events were identified in *N. lutea*, and 23 gene duplication events in *N. nucifera* (Fig. 4AB). These gene duplication events were all segmental duplication events, and no tandem duplication events occurred. The Ka and Ks of these gene duplication events were analyzed. The synonymous substitution rate distribution of the *NlbZIP* and *NnbZIP* duplication gene pairs were calculated. It was found that the Ks of the *NlbZIP* genes had a peak between 0.5 and 0.6. In contrast, the *NnbZIP* genes showed two peaks distributed at Ks between 0.4—0.5 and 0.6—0.7, respectively (Fig. 4C). The Ka/Ks ratios of all *bZIP*-replicated gene pairs were less than 1 (Fig. 4D). These results indicated that these genes underwent strong purification selection after fragment replication, and the function of these genes did not significantly differentiate.

**Fig. 5:**
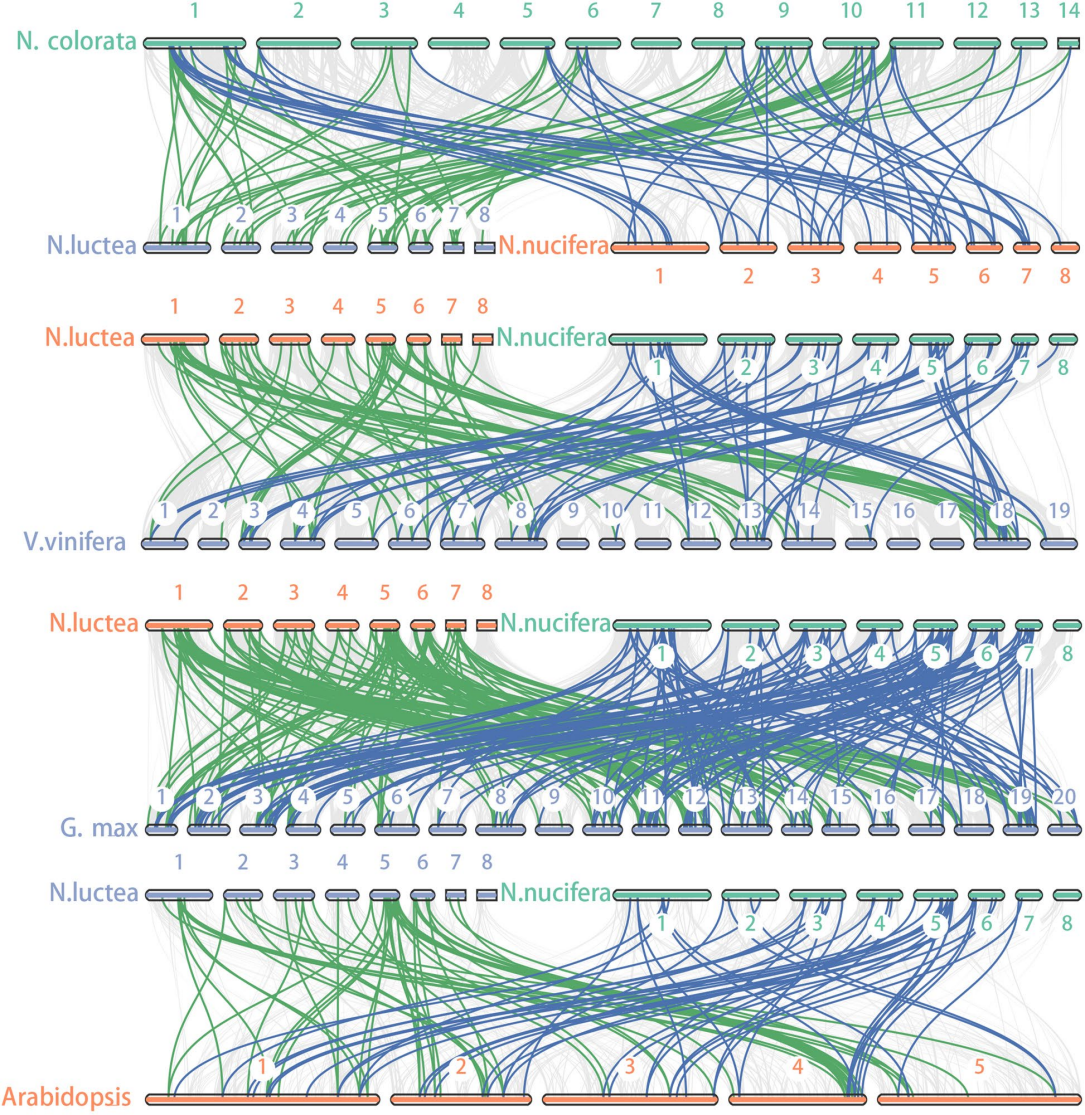
Synteny analysis of *bZIP* genes between two lotus species with *N.colorata*, *V. vinifera*, *G. Max*, and *Arabidopsis*. The green lines indicate covariance between *N. luctea* and the other four species, the blue lines indicate covariance between *N. nucifera* and the other four species

To further understand the evolution of the lotus *bZIP* gene family, the covariance of two lotus species with four other species (*Nymphaea colorata* (*N.colorata*), *Vitis vinifera* (*V.vinifera*), *Glycine max* (*G.max*) and *Arabidopsis*) were aligned using a python version of the MCScanX software (Fig. 5). Each of these four species represents an important point in the evolution of angiosperms. The results show that there is little difference in the number of co-linear pairs between the two lotus species and the *N.colorata* and *V.vinifera*. The number of homologous genes for *bZIP* was significantly increased on the *G.max* side of the lotus compared to *G.max*. The number of homologous genes on the *Arabidopsis* side was the lowest for these species. These results indicate that *bZIP* genes have experienced multiple expansion and contraction processes during the evolution of angiosperms, which may be the result of plants adapting to the changes in their natural environment.

### Alignment analysis of the *NnbZIP* genes with the *NlbZIP* genes

**Fig. 6:**
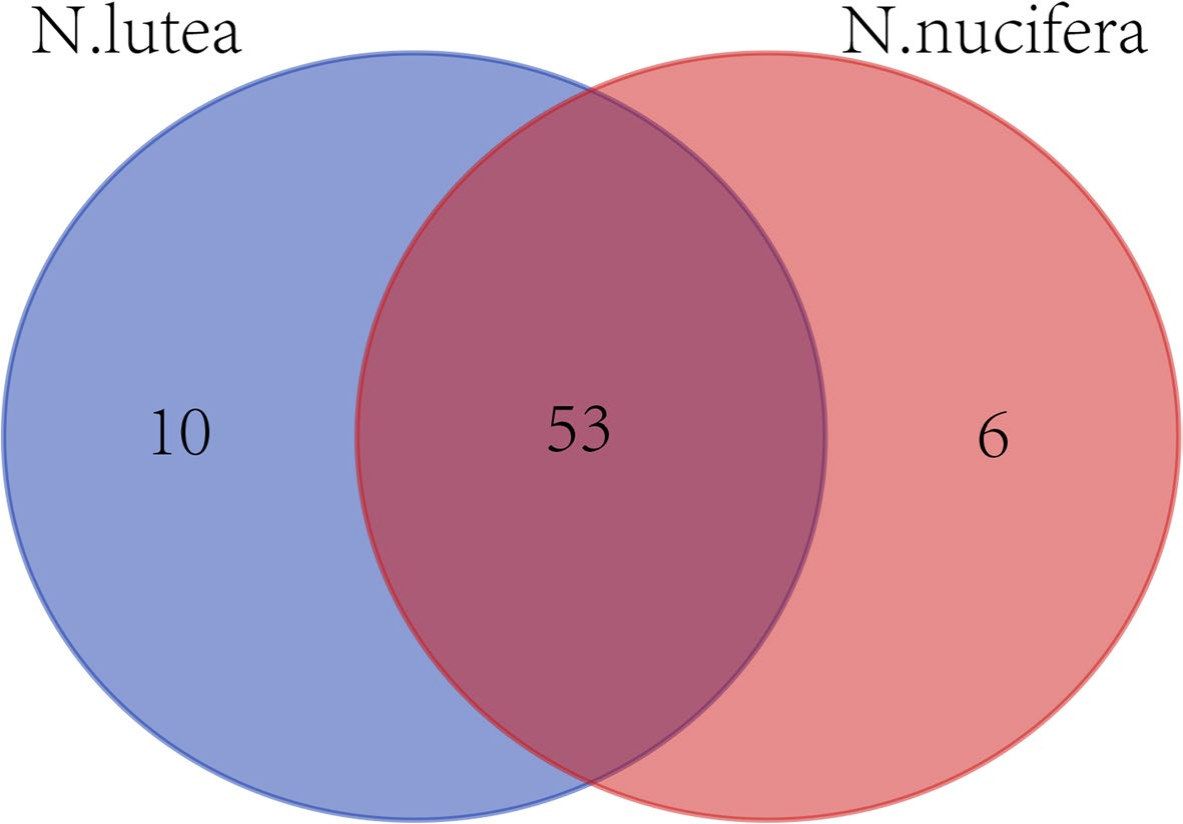
Venn diagram of homology between *N. luctea NlbZIPs* and *N. nucifera NnbZIPs*

The protein sequences of NnbZIPs and NlbZIPs were aligned using BLATp software. The results showed that the protein sequences of 53 *bZIP* genes were highly similar between *N. nucifera* and *N. lutea* (Fig. S3). There are 10 *bZIP* genes specifically present in *N. lutea* and 6 *bZIP* genes specific to *N. nucifera* (Fig. 6). This indicates that most of the functions of the *bZIP* gene family are similar between *N. nucifera* and *N. lutea*. The few unique *bZIP* genes may promote the formation of the characteristic traits of *N. nucifera* and *N. lutea.* This may be the result of the evolution of *N. nucifera* and *N. lutea* to adapt to the local environment. The gene information of unique to *N. nucifera* and *N. lutea* is represented in Table 2.

**Table 2:** The specifical *bZIP* genes in two lotus species

Species	Gene number	Gene name
*N. lutea*	10	NlbZIP03, NlbZIP13, NlbZIP18, NlbZIP19, NlbZIP20, NlbZIP24, NlbZIP49, NlbZIP51, NlbZIP60, NlbZIP65
*N. ucifera*	6	NnbZIP04, NnbZIP06, NnbZIP29, NnbZIP30, NnbZIP48, NnbZIP54

### The *bZIP* gene family involved in the regulation of anthocyanin biosynthesis in lotus

**Fig. 7:**
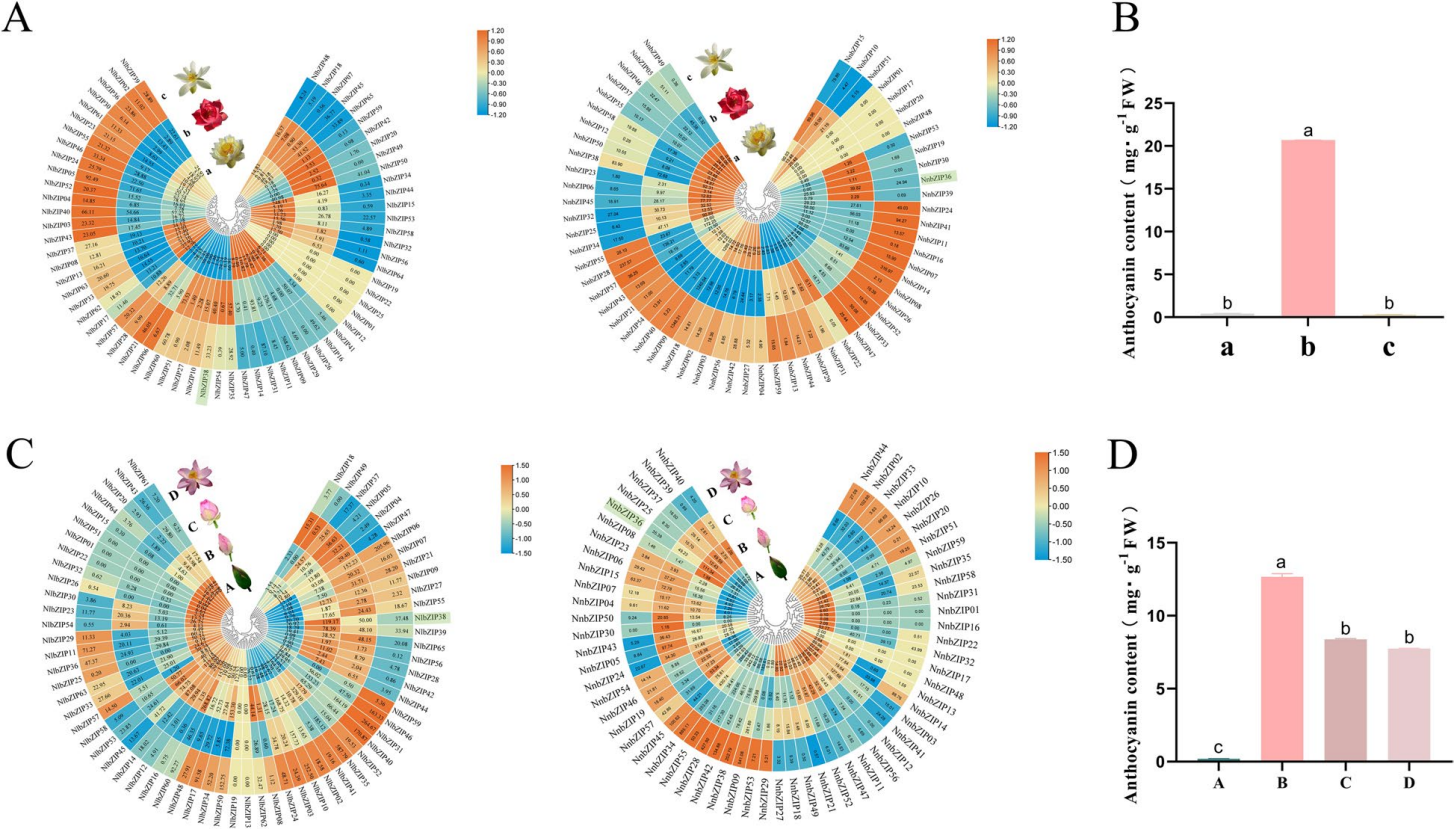
Potential ability of the *bZIP* genes to regulate anthocyanin synthesis in lotus. **A**: Expression profiling of the *bZIP* genes in lotus cultivars of different flower colors. **B**: Detection of anthocyanin content in different lotus cultivars. **C**: Expression profiling of the *bZIP* genes in different opening periods of red lotus cultivar. **D**: Detection of anthocyanin content in different opening periods of red lotus cultivar. (In the pictures, a indicates the lotus cultivar 'Jinsenianhua', b indicates the lotus cultivar 'Jinlinghuodu', c indicates 'Baiyinlian', A indicates the bud period of the ancient lotus, **B** indicates the late bud period of the ancient lotus, **C** indicates the early blooming period of the ancient lotus, and **D** indicates the full blooming period of the ancient lotus)

In order to understand the regulatory effect of *bZIP* gene family on lotus anthocyanins, the transcriptome data from three lotus cultivars with different flower colors were downloaded and reassembled. The anthocyanin content of three lotus cultivars with different flower colors were detected. The results showed that anthocyanin was only accumulated in red lotus (Fig. 7B), and the content of anthocyanin in red lotus was highest in late bud stage (Fig. 7D). Further transcriptome data showed that the individual *bZIP* gene members of *N. nucifera* and *N. lutea* did not seem to be directly related to the anthocyanin content of lotus. Interestingly, *NlbZIP38*, *NlbZIP50* and *NnbZIP36* were highly expressed in the red lotus cultivar 'Jinlinghuodu', while lower expressed in the white lotus 'Baiyinlian' and yellow lotus 'Jinsenianhua' (Fig. 7A). Further research found that the expression levels of *NlbZIP38* and *NnbZIP36* were consistent with the change trend of anthocyanin content in red lotus cultivar (Fig. 7C). This indicated that *NlbZIP38* and *NnbZIP36* may be involved in regulating the synthesis of lotus anthocyanins. The protein sequence comparison analysis showed that the protein sequences of NlbZIP38 and NnbZIP36 had a very high similarity (Fig. S4). NlbZIP38 and NnbZIP36 were considered to have the same function. Therefore, *NnbZIP36* was selected as a candidate gene for further verification in this paper.

### *NnbZIP36* promotes anthocyanin accumulation in *Arabidopsis*

**Fig. 8:**
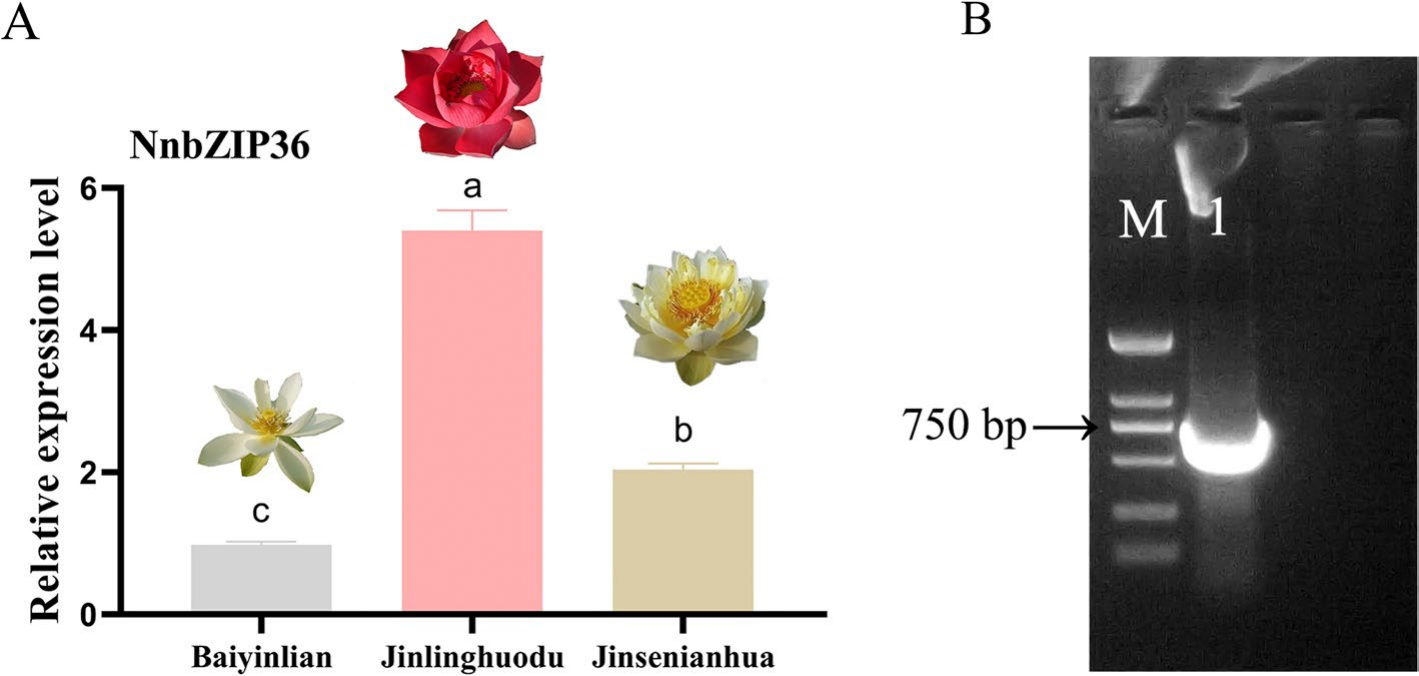
Expression analysis of *NnbZIP36* in different cultivars and cloning. **A**: Expression of *NnbZIP36* in the petals of yellow ‘Jinsenianhua’, red ‘Jinlinghuodu’ and white ‘Baiyinlian’ lotus cultivars by qRT-PCR. **B**: PCR amplification of NnbZIP36. M: DL ladder 2000 DNA marker. 1: NnbZIP36. Different letters indicate statistically significant differences compared to the corresponding ‘Baiyinlian’ (*p* < *0.05*)

**Fig. 9:**
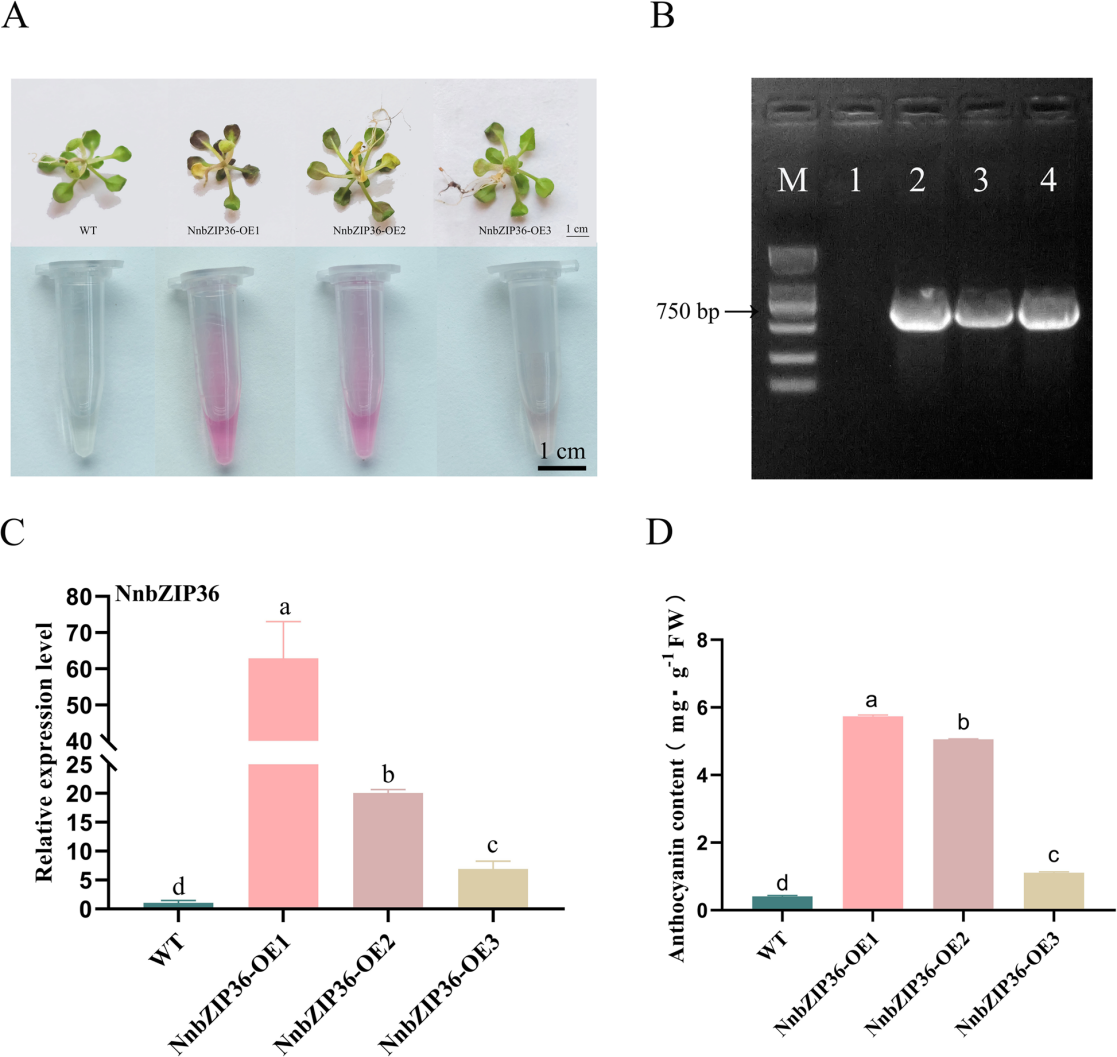
*NnbZIP36* overexpression promotes anthocyanin accumulation in *Arabidopsis.* **A**: Images of representative seedlings of wild-type and *NnbZIP36* overexpression lines. **B**: PCR identification of authentic *NnbZIP36* transgenic *Arabidopsis* plant lines. M: DL ladder 2000 DNA marker. 1: WT. 2: NnbZIP36-OE1. 2: NnbZIP36-OE2. 4: NnbZIP36-OE3. **C**: Expression analysis of *NnbZIP36* in wild-type and overexpressing *Arabidopsis* lines. **D**: *NnbZIP36* overexpression promotes anthocyanin accumulation in *Arabidopsis.* Different letters indicate statistically significant differences compared to the corresponding wild type (*p* < *0.05*)

**Fig. 10:**
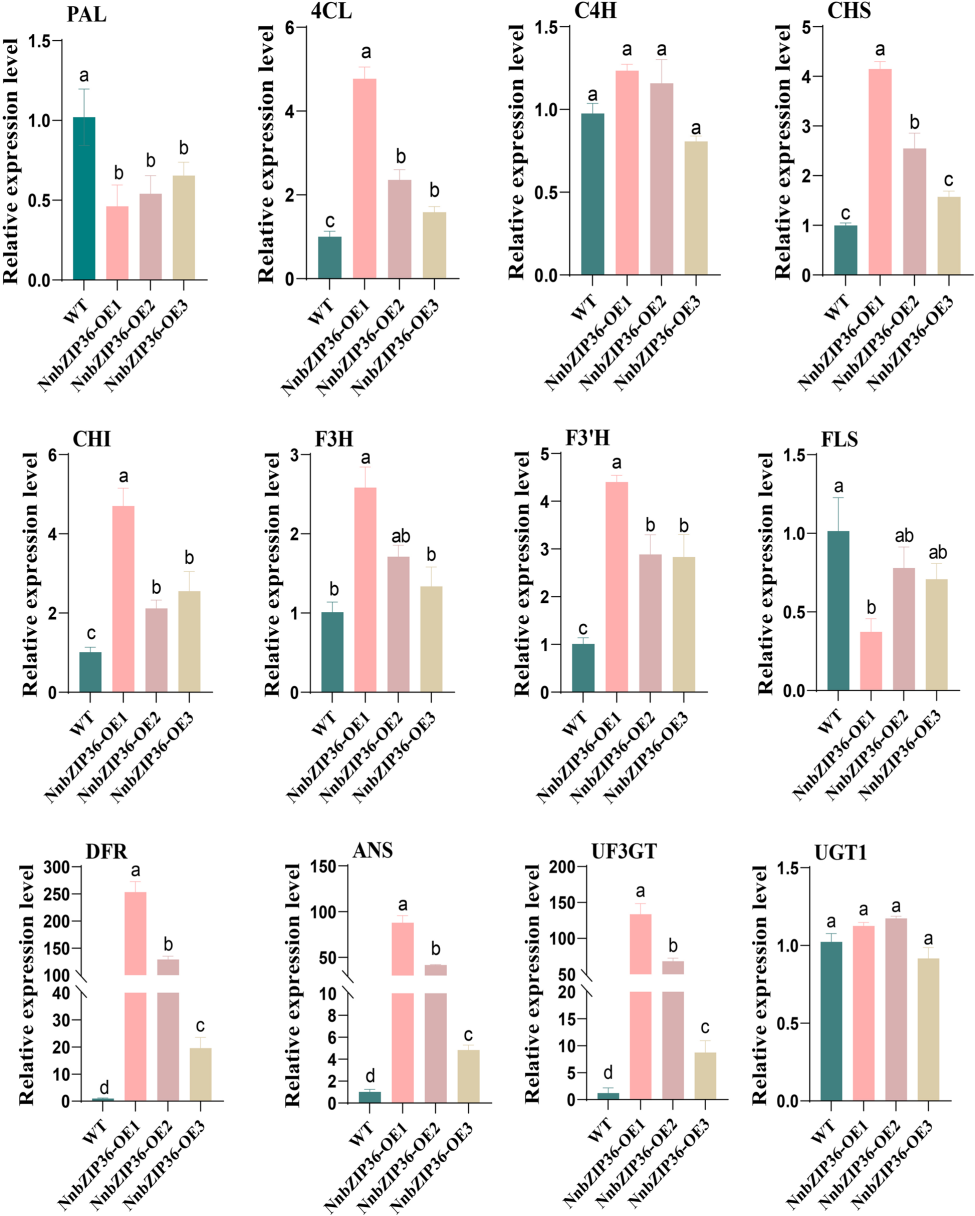
Expression analysis of anthocyanin synthesis-related genes in wild-type and *NnbZIP36* overexpressing *Arabidopsis* lines. The total RNA was extracted from leaves of different genotypes. Different letters indicate statistically significant differences compared to the corresponding wild type (*p* < *0.05*)

The result of qRT-PCR verified that *NnbZIP36* was only highly expressed in red lotus cultivar (Fig. 8A). To understand the regulation mechanism of *NnbZIP36* on anthocyanin synthesis, the open reading frame (ORF) of *NnbZIP36* gene was cloned (Fig. 8B). The pFAST-R05-NnbZIP36 overexpression vector was constructed. Three NnbZIP36-OE transgenic *Arabidopsis* lines were obtained by PCR and qRT-PCR experiments (Fig. 9BC). It was found that the petioles and leaves of the transgenic *Arabidopsis* were red (Fig. 9A). The anthocyanin content of NnbZIP36-OE *Arabidopsis* plants was significantly up-regulated (Fig. 9D). The expression of most structural genes (*4CL*, *CHI*, *CHS*, *F3H*, *F3'H*, *DFR*, *ANS* and *UF3GT*) on the anthocyanin biosynthetic pathway were significantly up-regulated in transgenic *Arabidopsis* compared with wild-type *Arabidopsis* (Fig. 10). These results showed that the *NnbZIP36* gene has a positive regulatory effect on the accumulation of anthocyanins in transgenic *Arabidopsis*.

## Discussion

bZIP TFs are a family of transcription factors that widely exist in eukaryotes and highly conserved in evolution [18]. As one of the largest transcription factor families, bZIP TFs play important roles in regulating plant growth, development and abiotic stress [7, 10, 30]. The *bZIP* family genes on a genome-wide scale have been systematically analyzed in many plant species, including some key crops, such as: *Arabidopsis* (79) [18], *Oryza sativa* (86) [19], *Vitis vinifera* (55) [52], *Solanum lycopersicum* (69) [21], *Actinidia chinensis* (81) [53], *Punica granatum* (65) [54], etc. However, until now, although the entire lotus genome has been sequenced [35, 36], the studies of *bZIP* gene family in lotus are limited. In this study, the *bZIP* gene family from genomes of two lotus species were identified and systematically analyzed. The potential functions of *bZIP* gene family were explored in the regulation of anthocyanin biosynthesis in lotus.

In this study, a total of 124 *bZIP* genes (59 *NnbZIPs* and 65 *NlbZIPs*) were identified in the genomes of two species of lotus (Table 1). It is similar to the number of *bZIP* gene family members in *Vitis vinifera* (55) [52], *Punica granatum* (65) [55] and other species. The *bZIP* genes in lotus were classified into 13 groups by constructing phylogenetic trees of the *bZIP* gene family in two species of lotus, *Ginkgo*, *Amborella*, *Nymphaea colorata, Vitis vinifera* and *Arabidopsis*, which is similar to the grouping of *Arabidopsis bZIP* gene family [18] (Fig. 1). In addition to the absence of *bZIP* gene members of *N. nucifera* and *Amborella* in group J. The *bZIP* gene members of seven species were distributed in the other 12 subgroups. This suggests that the number of *bZIP* genes is more conserved during evolution. Due to the highly conserved nature of bZIPs sequences, the *bZIP* genes in the same group have the same or similar functions, which provides a reference for studying the functions of this gene family [55].

Analysis of gene structure and conserved motifs can help us predict the function of genes [56]. It is also an important basis for studying gene evolution and gene replication [55]. The results of structural analysis of lotus *bZIP* genes showed that the *bZIP* gene structure of lotus is relatively simple, with the number of introns ranging from 0 to 11. It can be divided into two categories according to the presence or absence of introns. A total of 18 *bZIP* genes without introns were found in the genomes of two lotus species. Of these 18 *bZIP* genes, 15 were classified in group S and three *bZIP* genes (NnbZIP17, NnbZIP19 and NnbZIP54) were classified in group E. A similar situation exists in species such as *Arabidopsis*, strawberry and gourd [18, 52, 57]. *bZIP* genes located in different subgroups may play different roles in plant growth and development. For example, most members of group A are involved in the ABA biological pathway and regulate plant responses to abiotic stresses [58–60]. Genes located in group D can be involved in plant defense against pathogens [3]. Genes located in group G and their homologs are mainly involved in blue-violet signaling [61]. The lack of introns contributes to an accelerated post-transcriptional response to abiotic stresses [62]. Similar findings were observed in soybean and watermelon, revealing evolutionary imprinting and functional differentiation of genes associated with intron deletions [63–66]. The number of conserved motifs of each bZIP protein also varies greatly. There are many bZIP proteins with only 1 protein conserved motif, and the most have 6 protein conserved motifs (Fig. 2). It has been shown that the *bZIP* gene family has a variety of different conserved structural domains. In addition to the specific bZIP_1 (PF00170) and bZIP_2 (PF07716) domains, bZIP proteins in plants also have additional functionally conserved domains that participate in various biological processes [67]. In lotus, the identified bZIP members have three different conserved domains: BRLZ, bZIP_C and bZIP_2. Among them, the BRLZ conserved domain is widely present in the *bZIP* gene family of lotus. bZIP_C is found in 6 members (*NnbZIP26*, *NlbZIP29*, *NlbZIP02*, *NlbZIP18*, *NnbZIP27* and *NlbZIP30*), while bZIP_2 is only present in *NnbZIP17* (Fig. 2).

The *cis*-acting element of the promoter plays an important role in the transcription of the gene, its activity can directly affect the gene expression and function [68]. The function of the gene can be predicted according to the type of the *cis*-acting element in the gene promoter. Among the promoters of the 124 *bZIP* gene family members in lotus, there are generally more *cis*-acting elements related to stress resistance than those related to growth and development (Fig. 3). Implying that the *bZIP* gene family of lotus plays an important role in response to stress. A large number of response hormone-related elements (especially ABA response elements and MeJA response elements) also widely exist in the promoters of *bZIP* genes. Studies have shown that the transcription and regulation of many *bZIP* genes are induced by ABA [69]. Through ABA response elements (ABRE), *bZIP* genes are involved in coordinating responses to drought and other environmental factors as well as seed development processes [69]. It proves the role of *bZIP* gene family in regulating plant stress resistance. It is further shown that the *cis*-acting element of the promoter plays an indispensable role in the response of the *bZIP* gene to external environmental changes. In addition, a large number of MYB and MYC binding sites expanded the regulatory network of the *bZIP* gene family.

The expansion and contraction of gene families are the result of plant adaptation to different environmental conditions, while the tandem and fragment duplication events of gene families are crucial for the expansion of gene families and the diversification of gene functions [70]. The results of the MCScanX software analysis showed that the 24 pairs and 23 pairs of *bZIP* fragment duplication genes were detected in *N. lutea* and *N. nucifera*, respectively, and no tandem duplication events were detected in two lotus species (Fig. 4AB). The Ka/Ks ratios calculated for all gene pairs were less than 1 (Fig. 4D). This implies that these genes may have undergone strong purifying selection pressures during evolution and did not significantly alter the function of the *bZIP* genes. Furthermore, we compared the collinearity of the two lotus species with four other species (*N.colorata*, *V.vinifera*, *G.max* and *Arabidopsis*). The number of collinear pairs between the two kinds of lotus and *N.colorata* or *V.vinifera* is not much different. There are more collinear pairs between lotus and *G.max*, while the number of collinear pairs between lotus and *Arabidopsis* is greatly reduced (Fig. 5). This shows that lotus has a relatively close evolutionary relationship with *N.colorata* and *V.vinifera*. However, there is a relatively distant evolutionary relationship with *G.max* and *Arabidopsis*. It also indicated that the *bZIP* gene family experienced multiple expansion and contraction events during the evolution of angiosperms.

As a water-soluble pigment involved in the secondary metabolic pathway of plants, anthocyanins play an important role in providing bright colors to plants and helping them cope with the external environment [71]. In recent years, members of the *bZIP* gene family in many plants have been reported to be involved in the regulation of anthocyanins. For example, HY5 in *Arabidopsis* can promote the expression of genes related to anthocyanin synthesis. The accumulation of anthocyanins by binding to the promoter of MYB transcription factors or directly interacting with MYB transcription factors [10, 13, 30]. In apple, the bZIP transcription factor gene *MdHY5* can actively regulate the accumulation of anthocyanins by directly promoting the expression of *MdMYB10* and *MdMYB1* genes [29, 72, 73]. *RsbZIP011* and *RsbZIP102* were reported to be associated with anthocyanin content in radish [74]. *PgbZIP16* and *PgbZIP34* of pomegranate were overexpressed in *Arabidopsis* to promote anthocyanin content accumulation in *Arabidopsis* leaves [55]. VvbZIP36 was a negative regulator of anthocyanin synthesis in grapes and played a role in balancing the synthesis of stilbene (α-glucosin), lignans, flavonols, and anthocyanins [32]. These clues indicated that the *bZIP* gene family plays an important role in regulating anthocyanin synthesis and promoting anthocyanin accumulation in plants.

Anthocyanin is one of the main pigment substances that affect the flower color of lotus. Understanding the regulation mechanism of anthocyanin synthesis in lotus is of great significance for improving ornamental value and stress resistance [34]. In the present study, we found that *NnbZIP36* and its homologous gene *NlbZIP38* in lotus were significantly correlated with the anthocyanin content of lotus. The results of qRT-PCR further showed that *NnbZIP36* was highly expressed in red lotus cultivar of ‘Jinlinghuodu’. This implies that *NnbZIP36* has the ability to regulate the synthesis of anthocyanins in lotus. In order to study the function of *NnbZIP36*, we constructed the pFAST-R05-NnbZIP36 overexpression vector and transformed *Arabidopsis*. The results showed that the leaves and petioles of *Arabidopsis* overexpressed with *NnbZIP36* appeared red and the anthocyanin content was significantly increased (Fig. 9). Most genes in the anthocyanin synthesis pathway (*4CL*, *CHI*, *CHS*, *F3H*, *F3'H*, *DFR*, *ANS* and *UF3GT*) were significantly up-regulated in NnbZIP36 transgenic *Arabidopsis*. The expression level of *PAL* in *Arabidopsis* overexpressing *NnbZIP36* was lower than that of wild-type *Arabidopsis* (Fig. 10). This may be because *PAL* is a structural gene involved in the synthesis of several secondary metabolites but not directly involved in the synthesis of anthocyanins [75]. According to the basis of existing experimental studies, we have found that *NnbZIP36* can promote the anthocyanins synthesis in overexpressed *Arabidopsis*. However, the mechanism of *NnbZIP36* gene regulating anthocyanin synthesis in lotus needs further study. And the *NnbZIP36* interacts with other gene to co-regulate in flower color formation of lotus will be a new direction for our research.

## Conclusions

In this study, we identified a total of 124 *bZIP* genes (59 *NnbZIPs* and 65 *NlbZIPs*) from the genomes of two lotus species. The *bZIP* genes of lotus were divided into 13 groups by constructing the phylogenetic tree of seven species. Due to the high degree of conservation of the *bZIP* genes, proteins with similar functions were clustered into a group. The combination of gene structure, conserved motifs and promoter *cis*-element analysis provided a reliable basis for studying the functions of related genes in the lotus *bZIP* gene family. Candidate genes affecting anthocyanin biosynthesis were identified using transcriptome data analysis. The gene cloning, qRT-PCR analysis, and *Arabidopsis* transformation were performed. Our results suggested that *NnbZIP36* has a promoting role in anthocyanin accumulation. In our future studies, we will focus on the regulatory network comprising *NnbZIP36* and anthocyanin synthesis-related genes, and elucidate the molecular mechanism by which *NnbZIP36* interacts with other transcription factors to promote anthocyanin accumulation.

### Supplementary Information

**Additional file 1:** **Fig. S1.** Localization of the *bZIP* genes in the lotus genome. A: *N. nucifera*. B: *N. lutea*. **Fig. S2.** Distribution of cis-acting elements in the promoter region of *bZIP* genes in lotus. **Fig. S3.** The heat map of NnbZIPs and NlbZIPs protein sequence alignment rates. The different colored circles indicate the degree of protein sequence similarity. **Fig. S4.** Sequence alignment analysis of NnbZIP36 and NlbZIP38. **Table S1.** Sources of transcriptome data used in this study. **Table S2.** List of primers used in this study.

## Data Availability

The transcriptome data used in this article are available for download from NCBI (https://www.ncbi.nlm.nih.gov/sra/?term=). The Accession number used for the download is listed in Table S1. The genomic data of *Nelumbo nucifera* used in this article are available in the database of *Nelumbo* (http://nelumbo.cngb.org/nelumbo/home). The genomic data of *Nelumbo lutea* used in this article are available in the database of NCBI (https://www.ncbi.nlm.nih.gov/genome/9878).
