# Supplementary material for: Analysis of bZIP gene family in lotus (Nelumbo) and functional study of NnbZIP36 in regulating anthocyanin synthesis
Source: BMC Plant Biol. 2023 Sep 15;23:429. doi: 10.1186/s12870-023-04425-2 (PMC10503039; doi:10.1186/s12870-023-04425-2)
Supplement: Supplementary file 1 — Additional file 1: Fig. S1. Localization of the bZIP genes in the lotus genome. A: N. nucifera. B: N. lutea. Fig. S2. Distribution of cis-acting elements in the promoter region of bZIP genes in lotus. Fig. S3. The heat map of NnbZIPs and NlbZIPs protein sequence alignment rates. The different colored circles indicate the degree of protein sequence similarity. Fig. S4. Sequence alignment analysis of NnbZIP36 and NlbZIP38. Table S1. Sources of transcriptome data used in this study. Table S2. List of primers used in this study. [file 12870_2023_4425_MOESM1_ESM.docx]

| The following Supporting Information is available for this article:  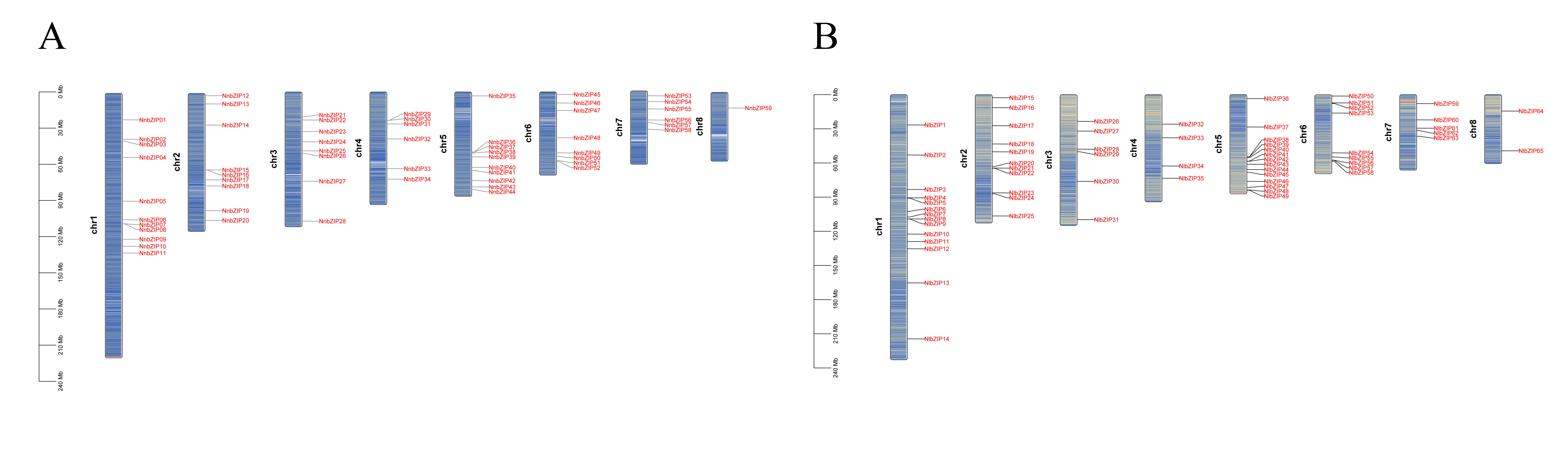 |
| --- |
| Fig. S1. Localization of the *bZIP* genes in the lotus genome. A: *N. nucifera*. B: *N. lutea*.   |
| Fig. S2. Distribution of cis-acting elements in the promoter region of *bZIP* genes in lotus.   |
| Fig. S3. The heat map of NnbZIPs and NlbZIPs protein sequence alignment rates. The different colored circles indicate the degree of protein sequence similarity.  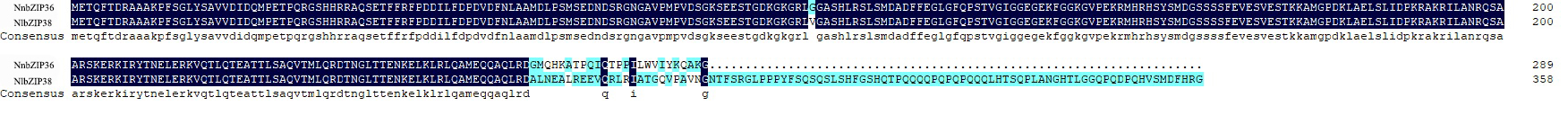 |
| Fig. S4. Sequence alignment analysis of NnbZIP36 and NlbZIP38. |
| Table S1 Sources of transcriptome data used in this study   \| **Data type** \| **Platform** \| **Cultivar Name** \| **Utility** \| **Tissue/developmental stage** \| **Accession number (NCBI SRA)** \| \| --- \| --- \| --- \| --- \| --- \| --- \| \| RNA \| Illumina \| Ancient Chinese lotus \| Gene expression \| full bloom stage (petal) \| SRR13024308 \| \| RNA \| Illumina \| Ancient Chinese lotus \| Gene expression \| initial flowering stage (petal) \| SRR13024309 \| \| RNA \| Illumina \| Ancient Chinese lotus \| Gene expression \| initial flowering stage (petal) \| SRR13024310 \| \| RNA \| Illumina \| Ancient Chinese lotus \| Gene expression \| initial flowering stage (petal) \| SRR13024311 \| \| RNA \| Illumina \| Ancient Chinese lotus \| Gene expression \| late flower bud stage (petal) \| SRR13024320 \| \| RNA \| Illumina \| Ancient Chinese lotus \| Gene expression \| initial flowering stage (petal) \| SRR13024331 \| \| RNA \| Illumina \| Ancient Chinese lotus \| Gene expression \| early flower bud stage (petal) \| SRR13024338 \| \| RNA \| Illumina \| Ancient Chinese lotus \| Gene expression \| early flower bud stage (petal) \| SRR13024343 \| \| RNA \| Illumina \| Ancient Chinese lotus \| Gene expression \| full bloom stage (petal) \| SRR13024352 \| \| RNA \| Illumina \| Ancient Chinese lotus \| Gene expression \| full bloom stage (petal) \| SRR13024353 \| \| RNA \| Illumina \| Ancient Chinese lotus \| Gene expression \| early flower bud stage (petal) \| SRR13024354 \| \| RNA \| Illumina \| Ancient Chinese lotus \| Gene expression \| early flower bud stage (petal) \| SRR13024355 \| \| RNA \| Illumina \| Baiyinlian \| Gene expression \| White FLowers (petal) \| SRR20242153 \| \| RNA \| Illumina \| Baiyinlian \| Gene expression \| White FLowers (petal) \| SRR20242167 \| \| RNA \| Illumina \| Baiyinlian \| Gene expression \| White FLowers (petal) \| SRR20242168 \| \| RNA \| Illumina \| Jinlinghuodu \| Gene expression \| Red FLowers (petal) \| SRR20242157 \| \| RNA \| Illumina \| Jinlinghuodu \| Gene expression \| Red FLowers (petal) \| SRR20242158 \| \| RNA \| Illumina \| Jinlinghuodu \| Gene expression \| Red FLowers (petal) \| SRR20242159 \| \| RNA \| Illumina \| Jinsenianhua \| Gene expression \| Yellow FLowers (petal) \| SRR20242164 \| \| RNA \| Illumina \| Jinsenianhua \| Gene expression \| Yellow FLowers (petal) \| SRR20242165 \| \| RNA \| Illumina \| Jinsenianhua \| Gene expression \| Yellow FLowers (petal) \| SRR20242166 \| |
| Table S2 List of primers used in this study   \| **Gene name** \| **Primer sequence** \| **Usage** \| \| --- \| --- \| --- \| \| NnbZIP36 \| F: GCGATGGAAACCCAGTTCAC \| Gene cloning \| \| R: GCCTCAACCTTTCGCTTGTTTGTA \| Gene cloning \| \| NnbZIP36 \| F: TCACTATGGCGGCCCCTCGAGGCGATGG  AAACCCAGTTCAC \| Vector construction \| \| R: TATCCAGTCACTATGGTCGACGCCTCAAC  CTTTCGCTTGTTTGTA \| Vector construction \| \| NnbZIP36 \| F: CATTTGAGGTGGAGTCCGTG \| qRT-PCR \| \| R: ACCTTCCTCTCTAGCTCGTTGGT \| qRT-PCR \| \| NnActin \| F: GCGTTCTGCCGTCTTCTAAA \| qRT-PCR \| \| R: CCCTCTTGGATTGTGCCTC \| qRT-PCR \| \| At4CL \| F: CGCAAACCCTTTCTACACT \| qRT-PCR \| \| R: CAATTCTCCGGTGTTGGTTC \| qRT-PCR \| \| AtC4H \| F: CCTCCATGATGCGAAGCTC \| qRT-PCR \| \| R: GCTTCGACGTCCAACACC \| qRT-PCR \| \| AtPAL \| F: GGCCAATTCCGCGACA \| qRT-PCR \| \| R: ATTTAGCTCATCGCGGACA \| qRT-PCR \| \| AtFLS \| F: AGAGACCAGCACAAGCC \| qRT-PCR \| \| R: GCCACTTTACGCGCCAC \| qRT-PCR \| \| AtCHS \| F: CGTCTTCTGCACTACCTCC \| qRT-PCR \| \| R: TGGTACATCATGAGACGCTT \| qRT-PCR \| \| AtCHI \| F: CCATCTCCAAACGCGAAA \| qRT-PCR \| \| R: AGCCAATAAGTTCTCGTCCA \| qRT-PCR \| \| AtF3H \| F: GTGTTTAGCGACGAAATCCC \| qRT-PCR \| \| R: TCTCACAAGCCTCAACGA \| qRT-PCR \| \| AtF3’H \| F: ACTAAGCCTCATCGAACCC \| qRT-PCR \| \| R: ATGCCATGTGTTTAGCTCC \| qRT-PCR \| \| AtDFR \| F: TTGTTCGTGCCACCGTTC \| qRT-PCR \| \| R: TCCATTCACTGTCGGCTT \| qRT-PCR \| \| AtANS \| F: TCTCTCTGTCGGTCTAGGTT \| qRT-PCR \| \| R: AGTATCCCCAATGTGCATC \| qRT-PCR \| \| AtUF3GT \| F: ATGTATCCGTGGTTAGCCTT \| qRT-PCR \| \| R: AGTTTGTCTCCGCACCC \| qRT-PCR \| \| AtUGT1 \| F: TCAACCGACGAACGCGAA \| qRT-PCR \| \| R: CCTCAACTCCACCGACT \| qRT-PCR \| \| AtActin2 \| F: AGATGCCCAGAAGTCTTGTTCC \| qRT-PCR \| \| R: TTTGCTCATACGGTCAGCGATA \| qRT-PCR \| |
